# A functional connectome: regulation of Wnt/TCF-dependent transcription by pairs of pathway activators

**DOI:** 10.1186/s12943-015-0475-1

**Published:** 2015-12-08

**Authors:** Jamie Freeman, David Smith, Branko Latinkic, Ken Ewan, Lee Samuel, Massimo Zollo, Natascia Marino, Lorraine Tyas, Nick Jones, Trevor C. Dale

**Affiliations:** School of Biosciences, Cardiff University, Museum Avenue, Cardiff, CF10 3AX Wales UK; Faculty of Epidemiology and Population Health, London School of Hygiene and Tropical Medicine, Keppel Street, London, WC1E 7HT UK; Department of Mathematics, Imperial College, London, SW7 2AZ UK; Department of Molecular Medicine and Biotechnology and Centro di Ingegneria Genetica e Biotecnologia Avanzate, Federico II, Via Pansini 5, 80131 Naples, Italy

**Keywords:** Wnt signaling, Network, Protein complexes, Drug discovery, Cancer

## Abstract

**Background:**

Wnt/β-catenin signaling is often portrayed as a simple pathway that is initiated by Wnt ligand at the cell surface leading, via linear series of interactions between ‘core pathway’ members, to the induction of nuclear transcription from genes flanked by β-catenin/TCF transcription factor binding sites. Wnt/β-catenin signaling is also regulated by a much larger set of ‘non-core regulators’. However the relationship between ‘non-core regulators’ is currently not well understood. Aberrant activation of the pathway has been shown to drive tumorgenesis in a number of different tissues.

**Methods:**

Mammalian cells engineered to have a partially-active level of Wnt/β-catenin signaling were screened by transfection for proteins that up or down-regulated a mid-level of TCF-dependent transcription induced by transient expression of an activated LRP6 Wnt co-receptor (∆NLRP).

**Results:**

141 novel regulators of TCF-dependent transcription were identified. Surprisingly, when tested without ∆NLRP activation, most up-regulators failed to alter TCF-dependent transcription. However, when expressed in pairs, 27 % (466/1170) functionally interacted to alter levels of TCF-dependent transcription. When proteins were displayed as nodes connected by their ability to co-operate in the regulation of TCF-dependent transcription, a network of functional interactions was revealed. In this network, ‘core pathway’ components (Eg. β-catenin, GSK-3, Dsh) were found to be the most highly connected nodes. Activation of different nodes in this network impacted on the sensitivity to Wnt pathway small molecule antagonists.

**Conclusions:**

The ‘functional connectome’ identified here strongly supports an alternative model of the Wnt pathway as a complex context-dependent network. The network further suggests that mutational activation of highly connected Wnt signaling nodes predisposed cells to further context-dependent alterations in levels of TCF-dependent transcription that may be important during tumor progression and treatment.

**Electronic supplementary material:**

The online version of this article (doi:10.1186/s12943-015-0475-1) contains supplementary material, which is available to authorized users.

## Background

Wnt signaling plays a crucial role in normal development and disease. Deregulated Wnt signaling has been implicated in cancers arising in many different tissues [1]. Wnt ligand binding to Fz/LRP co-receptors inhibits β-catenin turnover leading to the activation of β-catenin/TCF-dependent transcription and target genes including c-myc [2, 3]. In colon cancer, mutations in pathway components have been identified in the majority of tumours [1, 3–5]. Enhanced Wnt signaling drives increased cell proliferation, decreased differentiation and the formation of adenomas [4, 6]. In breast cancer, mutations to pathway components are rare despite the observation that β-catenin is aberrantly stabilised in over 50 % of tumours. Epigenetic changes leading to altered expression of Wnt pathway regulators has been suggested to drive tumorigenesis in cancers lacking well defined mutations [6–8].

The classic Wnt signaling pathway can be presented as a simple linear pathway involving the following ‘core’ components: Wnt/Fz/LRP→Dsh--|Axin/APC/GSK-3--|β-catenin→TCF. However, this linear view does not convey the degree of complexity suggested by screens in *D. melanogaster*, HeLa and colon cancer cells that have identified over 250 genes as regulators of TCF dependent transcription [7, 9–12]. In many cases the mechanism(s) by which newly-identified regulators interact with ‘core’ pathway components is unknown. This may, in part, be because their mechanism(s) of action are context-dependent [9, 11–14]. Given the number of Wnt/β-catenin pathway regulators that have been identified in screens, a key challenge is the prediction of whether any particular gene product will regulate Wnt signaling in each of multiple potential cell contexts.

This study describes a screen that identified known and novel cDNAs that up- and down-regulated TCF-dependent transcription. Pair-wise combinations of genes were found to synergise in their induction of TCF-dependent transcription and large-scale mapping of these synergistic interactions uncovered a network of functional interactions. The structure of the Wnt network highlights potential mechanisms of functional synergy in cancer cells and can be used to identify novel points for therapeutic intervention.

## Results and discussion

**Fig. 1:**
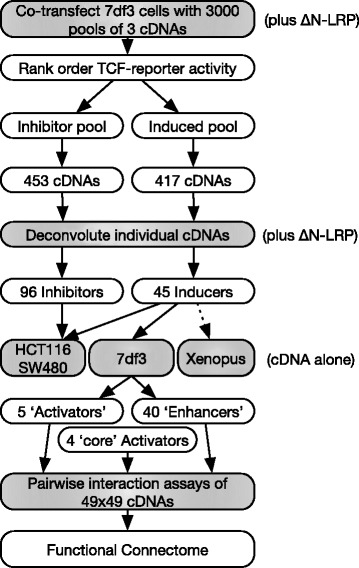
A schematic overview of the screening and pairwise assays

**Fig. 2:**
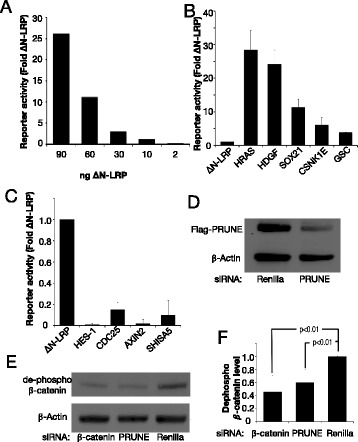
Identification of novel Wnt regulators. **a** Dose-dependent induction of TCF dependent transcription in 7df3 cells by constitutively active LRP6 (∆NLRP). **b** and **c** Examples of the 45 inducers (**b**) and 96 inhibitors (**c**) of TCF-dependent transcription identified from a cDNA library screen of 9000 *Xenopus tropicalis* cDNAs (see Additional file 1: Figure S1). **d** Prune siRNA reduced h-Prune protein levels. FLAG-tagged h-Prune was transfected into MDA-MB231 breast cancer cells 24 h after transfection of either Renilla luciferase or Prune siRNA. Expression was detected with an anti-FLAG antibody. **e** Knockdown of Prune by siRNA reduced the level of active (de-phosphorylated) β-catenin in MDA-MB231 cells. Blot shown is representative of four separate experiments. **f** Levels of active β-catenin after siRNA transfection in 4 separate experiments were quantified. Knockdown of Prune reduces levels of de-phosphorylated β-catenin to a level that was not significantly different from the knockdown of β-catenin (Student’s *T*-test, *p* < 0.05)

To identify novel regulators of Wnt signaling, a highly-inducible HEK293 based TCF-luciferase reporter cell line, with low levels of basal Wnt activity (7df3, [6–8]), was used to screen an expression library of 9000 full-length cDNAs from *Xenopus tropicalis* [7, 9–12]. An overview of this and subsequent experiments is shown in Fig. 1. To identify both positive and negative regulators, a constitutively active form of the Wnt LRP6 co-receptor (ΔNLRP), which induced a mid-level of transcription (~15-fold activation; Fig. 2a), was co-transfected with each pool of 3 cDNAs (3000 pools of 3 cDNAs). This approach allowed the identification of modulators that could contribute to a ‘just right’ level of Wnt pathway activity as found in tumours [15]. Luciferase reporter activity was normalised to expression from a co-transfected CMV-LacZ plasmid. A set of 151 inhibitor and 139 inducer cDNA pools were selected based on a combination of their fold induction/repression and their variation from the plate mean (Additional file 1: Figure S1). Assaying the individual cDNAs from hit pools identified 45 inducers and 96 inhibitors (example inducers and inhibitors are shown in Fig. 2b, c and a full list is presented in Additional file 2: Table S1). No correlation between CMV-LacZ expression and luciferase activity was observed, suggesting that cDNAs did not affect general transcription. cDNAs encoding the known Wnt pathway modulators CK1ε, CK1δ, Dvl2 and Axin2 were identified, confirming the screen identified Wnt regulators.

One of the strongest inducers (12.7 fold; Additional file 2: Table S1) was the *Xenopus Tropicalis* cDNA for the gene Prune. When assayed in the cognate *Xenopus laevis* animal cap explant system, Prune induced expression of Siamois, a classic Wnt/β-catenin target. In addition, Prune induced partial axis duplication in ventrally injected embryos (Additional file 3: Table S2), a phenotype that is consistent with the activation of the Wnt signaling pathway in *X. laevis*. PRUNE is amplified and overexpressed in breast cancer [9, 11–14, 16] while Wnt/β-catenin signaling is particularly activated in triple negative breast cancer (TNBC) [13, 17]. To determine if Prune was required for Wnt signal transduction we reduced PRUNE levels in the TNBC breast cancer cell line MDA-MB231 by siRNA transfection. MDA-MB231 cells have raised levels of endogenous β-catenin [18] and knock down of PRUNE led to a 40 % decrease of active (i.e. unphosphorylated) β-catenin levels that was equivalent to that induced by an siRNA to β-catenin itself (Fig. 2d-f). Taken together the data suggest that Prune may regulate Wnt/β-catenin signaling in an oncogenic context.

### Wnt pathway activators

**Fig. 3:**
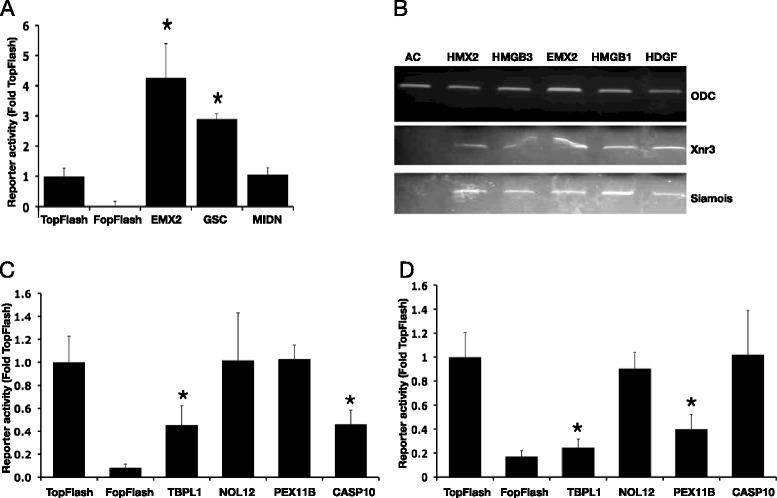
Transcription regulation in colon cancer cells and *Xenopus laevis*. **a** Example ‘inducer’ cDNAs effect on TopFlash reporter when co-transfected into HCT116 cells (see Additional file 4: Table S3 for complete list). Comparison with the mutant FopFlash reporter demonstrates high basal levels of β-catenin/TCF-dependent transcription. Results are from triplicate wells across different plates. Stars represent statistical significantly different values from TopFlash (Benjamini-Hochberg corrected *p* < 0.01). **b** Expression of a subset of ‘inducers’ activated transcription of the endogenous Wnt target genes Xnr3 and Siamois in animal cap assays. AC; uninjected animal caps. ODC; ornithine de-carboxylase control. **c** and **d** TopFlash reporter activity from a subset of cDNAs co-transfected into SW480 cells (**c**) or HCT116 cells (**d**). (see Additional file 3: Table S2 for complete a list). The data shown are from triplicate wells across different plates. Stars represent statistically significantly different values from TopFlash (Benjamini-Hochberg corrected *p* < 0.01)

To further validate function, the activators were expressed in HCT116 colon cancer cells that contain an endogenous activated β-catenin allele, significant proportions of which are present in membrane-bound complexes [19], and show a mid-level of TCF-reporter activity as determined by co-transfection with the TOPflash plasmid. After using the Benjamini-Hochberg method [20, 21] to correct for multiple hypothesis testing, 14 of the 45 activators were found to further increase (super-activate) reporter levels (Benjamini-Hochberg corrected *p*-values ranged from *p* = 3.4 × 10^−3^ to *p* = 6.6 × 10^−28^; Fig. 3a, Additional file 4: Table S3). Thus the ability to super-activate transcription was shared by a significant subset of genes initially identified in the HEK293-based reporter cell line.

A subset of the strongest activators were assayed in *X. laevis* secondary axis induction experiments, and for the ability to activate the Wnt target genes Xnr3 and Siamois in a *X. laevis* animal cap assays. RNAs that induced the formation of a complete secondary axis (HMX2, HMGB3, HRAS, EMX2, HMGB1, ZNF616, and HDGF) also strongly induced expression of Wnt target genes (Fig. 3b, Additional file 3: Table S2). Interestingly, HMGB1 and HMGB2 have previously been linked to altered Wnt signaling in cartilage development, providing further support linking the set of genes to Wnt signalling [2, 22].

### Wnt pathway inhibitors

The 96 inhibitory cDNAs identified in the screen were assessed for their effects in two different colon cancer lines. When transfected into SW480 cells that have high levels of ‘active’ b-catenin and highly active TCF dependent transcription following APC deletion, nearly half (42/96) of the inhibitors reduced TCF-dependent transcription (Benjamini-Hochberg corrected *p* < 0.01; Additional file 4: Table S3, Fig. 3c; [23]). By contrast, only 15/96 cDNAs showed significant inhibition of TCF-dependent transcription in HCT116 cells that contain mutant, constitutively active β-catenin (Benjamini-Hochberg corrected *p* < 0.01; Additional file 4: Table S3, Fig. 3d). Eight of the inhibitors were active in both cell lines, and 4 were able to inhibit TCF-dependent transcription by greater than 50 % in both cell lines (TBPL1, IDH2, SFRS3, RXRβ). The distinct responses to inhibitor expression in the different lines suggested a context dependence of cellular responses.

### Functional interactions between cDNA pairs

**Fig. 4:**
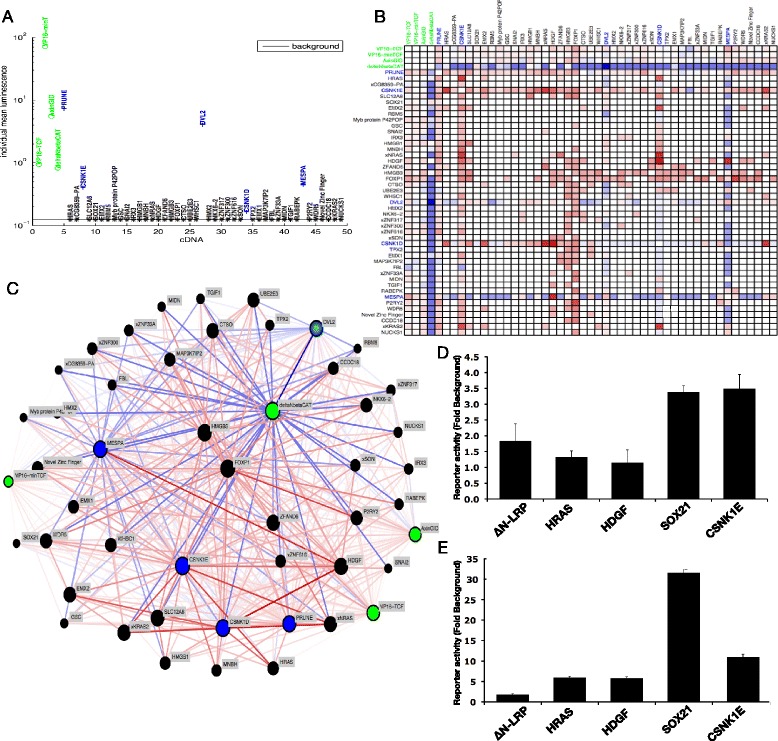
A network of functional interactions that modulate TCF-dependent transcription. **a** Reporter activity of cDNAs individually transfected into 7df3 cells. Green circles represent constitutively active versions of ‘core’ pathway members. The 5/45 cDNAs that were able to activate TCF dependent transcription to a level greater than background (Kolmogorov-Smirnov test, *p* < 0.01) are shown in blue. All other cDNAs were unable to activate TCF dependent transcription when individually transfected and are shown in black. ΔNLRP levels of activation were defined as 10^0^. **b** Heat map displaying functional interactions. Red and blue squares indicate positive or negative interaction respectively. The opacity of the colour indicates the strength of the interaction. **c** Network visualisation of functional interactions. Positive (*red*) and suppressive (*blue*) interactions whose strength is represented by line opacity are displayed. Core nodes are coloured green, activator nodes are blue whilst enhancers are black. The core pathway members tend to be the most highly connected nodes. **d** and **e** Synergistic interactions were observed using promoter-reporters driven by the endogenous Wnt target genes Myc (**d**) or Siamois (**e**) in HEK293 cells. All combinations with the exception of Ras/CK1δ and the Siamois promoter showed (multiplicative) synergistic responses

To determine the capacity of the 45 activators to increase TCF dependent transcription independently of ΔNLRP, they were individually transfected into the 7df3 reporter cells (Fig. 4a; Additional file 5: Table S4). Surprisingly, 40/45 failed to activate TCF-dependent transcription when assessed alone ((K-S) test, *p* < 0.01). Five cDNAs (Prune homolog (PRUNE2), Dishevelled (DVL2), Casein kinase 1ε (CSNK1E), Mesoderm posterior (MESPA) and Casein kinase 1δ (CSNK1D)) induced TCF-dependent transcription above background levels (Fig. 4a). Four of these activators satisfy a Benjamini and Hochberg corrected *p*-value of <0.05, whereas the final activator identified using an uncorrected *p*-value of <0.01 (CSNKD) had a corrected *p*-value of 0.052. Dishevelled and Casein kinases 1ε and δ have previously been implicated in the modulation of Wnt signaling [24].

Up until this point, any cDNA identified through its ability to super-activate TCF-dependent transcription was described as an inducer. From this point on, cDNAs that individually activated TCF-dependent transcription will be referred to as *activators*, while those that required the co-expression of other cDNAs such as ΔNLRP for their activity to be apparent will be identified as *enhancers*.

The conditional dependence of 40 out of the initial 45 activating cDNAs on ∆NLRP expression raised the question as to whether their ability to enhance TCF-dependent transcription was specific to ∆NLRP or whether they could enhance TCF-dependent transcription in combination with other activators or enhancers. To explore this question, the 40 enhancers and 5 activators identified in the screen were co-transfected with each other and with a further 4 constitutive ‘core’ activators (Fig. 1). The additional ‘core activators’ of Wnt signaling comprised a form of β-catenin lacking the N-terminal phosphorylation domain (∆NβCAT), the GSK-3 binding domain of Axin (AxinGID), a VP16 transactivation domain fused to full length TCF4 (VP16-TCF) and a VP16 transactivation domain fused to the minimal HMG box DNA binding domain of TCF (VP16-minTCF) [8]. In total 1170 pairwise combinations were assayed for synergy. As some ‘core’ activators were individually capable of inducing high levels of TCF-dependent transcription, the amounts of the ‘core’ activator’s cDNAs were reduced to induce a mid-range induction of reporter activity (Fig. 4a; ΔNLRP-induced levels were defined as 10^0^).

The 1170 combinations were assessed to identify if they were significantly different from background (K-S test; *p* < 0.01) before a weight was assigned to each combination. The weight quantifies the experimental synergy between two co-transfected cDNAs by comparison with the reporter activity induced by each cDNA of the pair when expressed alone, as described in Methods. Using a cut-off that satisfies a Benjamini and Hochberg corrected *p*-value of <0.03, 315/1170 pairwise cDNA combinations showed significantly higher levels of TCF-dependent transcription and 151 showed significantly lower levels than expected if there were no functional interaction (Fig. 4b, c). None of the synergistic interactions correlated with changes in expression from the co-transfected CMV driven LacZ reporter, suggesting that the functional interactions were specific for β-catenin/TCF-dependent transcription. The results were displayed as a heat map and as a network in which cDNAs were depicted as nodes and synergy as links (Fig. 4b, c). Synergistic interactions were very common, but were not evenly distributed. In particular, activator nodes including ‘core’ pathway components were most highly connected (Additional file 6: Figure S2). Synergistic interactions between cDNA pairs were also observed in the regulation of the Wnt target promoters c-Myc and Siamois, suggesting that functional interaction was not restricted to reporters containing artificial multimerized TCF-binding elements (Fig. 4d, e).

**Fig. 5:**
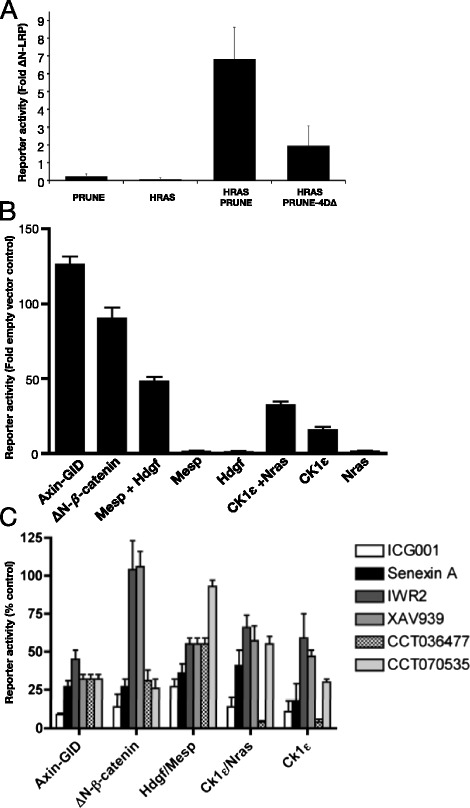
Functional interrogation of interacting pairs. **a** h-Prune synergy with HRAS was reduced following mutation of h-Prune catalytic residues (Prune 4Ddelta) [40]. Activation by cDNAs pairs (**b**) and inhibition by anti-Wnt signaling compounds (**c**) Compounds were added at 5 times their IC50 48 h after transfection. Luciferase assays were carried out 24 h later and were normalised to CMV-lacZ levels

The *Xenopus* PRUNE phosphodiesterase was the strongest novel activator in the absence of ΔNLRP (Fig. 4a). By contrast, human PRUNE, perhaps due to its use at its physiological temperature was incapable of activating TCF-dependent transcription (Fig. 5a). However, when human PRUNE was assessed in combination, it synergised with 19/45 cDNAs. These included both CSNK1D and CSNK1E which have previously been linked to Prune and its direct binding partner, the metastasis suppressor nm23-H1 (Fig. 5a, Additional file 7: Figure S3) [25]. PRUNE’s phosphodiesterase activity has been implicated as a target for the tumour progression phenotype associated with ASAP1 [26]. Point mutations within human PRUNE’s cAMP phosphodiesterase domain resulted in lower levels of TCF-dependent transcription suggesting that functional interaction relied at least in part on the phosphodiesterase activity (Fig. 5a).

### Using the functional connectome to study drug action

The pairs of inducers of β-catenin/TCF-dependent transcription should allow therapeutic agents to be tested in a defined cellular background (7df3 cells) against regulator combinations that would normally be activated in specific cellular contexts such as cancer cells. To explore this potential, known activators as well as two cDNA pairs (HDGF/MESP, CSNK1E/KRAS) were transfected into 7df3 cells and treated with a number of compounds for 24 h prior to luciferase reporter assay (Fig. 5b, c). ICG001 is known to interfere with β-catenin-CBP interactions (required for transcriptional transactivation) [27], XAV939 and IWR-2 promote β-catenin degradation by stabilising Axin [28], Senexin A inhibits the kinase CDK8 [29] while the molecular target(s) of CCT036477 and CCT070535 have not yet been identified [8]. TCF-dependent transcription induced by the cDNA combinations showed different sensitivity to inhibition by the small molecules (Fig. 5b, c). Transcription induced by the CSKN1E/NRAS and CSKN1E alone was fully inhibited by CCT036477 when used at five times the concentration required to block transcription induced by an activated Dvl2-ER fusion protein [8]. By contrast, this combination was less affected by CCT70535. Other activating pairs such as the HDGF/MESPA combination was largely resistant to inhibition by both CCT036477 and CCT70535, suggesting that the expression of HDGF in a subset of colon cancers may be a negative prognostic biomarker for CCT070535-related therapies [30]. ICG001 showed very broad efficacy, while XAV939 and IWR-2 were ineffective against mutant β-catenin as would be expected from their mechanism of action; both showed partial efficacy against other cDNA combinations. Taken together, these studies suggest that combinations of activators and enhancers might in future help predict biomarkers whose overexpression confers sensitivity or resistance to Wnt pathway therapeutic agents.

### Functional interactions in colon cancer cells

**Fig. 6:**
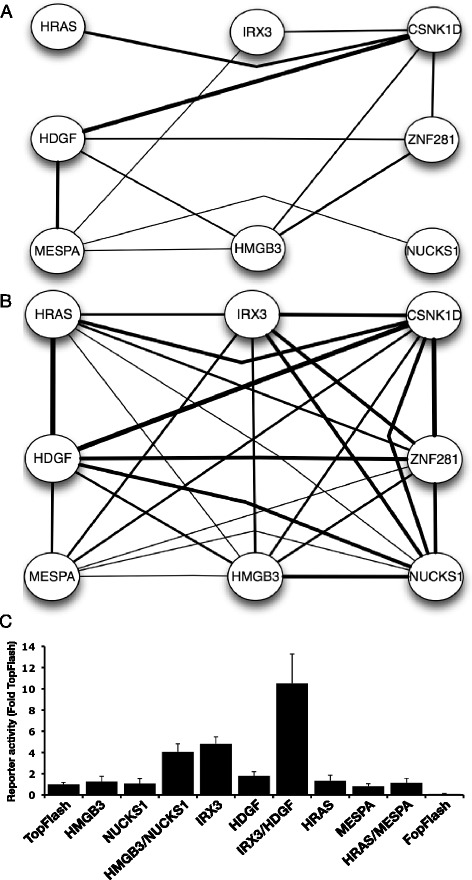
Comparison of functional network structure in 7df3 and HCT116 cells. **a** Pattern of functional interaction between a subset of cDNAs in 7df3 TCF-luciferase reporter cells. The thickness of the line represents the relative strength of the synergy. **b** Pattern of functional interaction in HCT116 cells. Functional interaction between cDNAs is indicated by a line between the relevant nodes when the activity of cDNA pairs exceeded the product of the reporter activity for each cDNA when expressed individually (Student’s *t*-test, *p* < 0.01). **c** Functional interaction between HMGB3/NUCKS1, and IRX3/HDGF in HCT116 cells

To investigate whether the network of functional interactions identified in the 7df3 cells was observed in other cell types a subset of cDNA nodes identified in the 7df3 reporter cells was examined for functional interaction in HCT116 cells (Fig. 6). HCT116 cells were transiently co-transfected with pair-wise combinations of 8 cDNAs together with the TopFlash reporter. 26/28 total possible interactions were detected in HCT116 cells (Fig. 6b, c). This number comprised many more functional interactions than observed between the same cDNAs in the 7df3 reporter line (Fig. 6a). Some cDNA pairs did not functionally cooperate in either 7df3 or HCT116 cells (Eg. HRAS-MESPA) suggesting that underlying mechanisms of functional interaction may be conserved.

### Comparison of sets of Wnt regulators

**Fig. 7:**
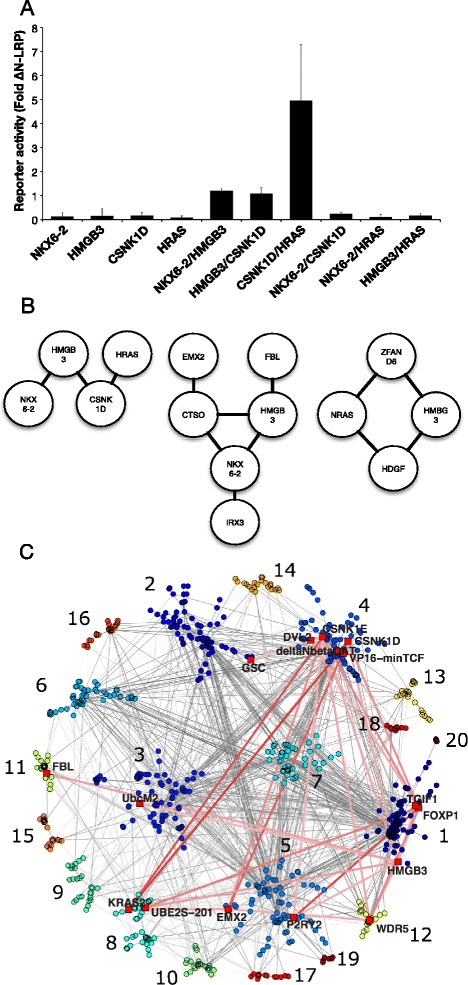
Non-overlapping interaction patterns and protein interaction networks. **a** Data from a subset of the 7df3 network displayed in more detail to highlight an important feature of the network; that functional co-operativity is limited to selected enhancer combinations. **b** Further examples of simple non-overlapping sub-networks. **c** Overlay of the ‘functional connectome’ on a Wnt PIN. Twenty protein communities (coloured nodes with grey links indicating protein interaction) were identified by modularity maximisation using the Louvain method. To guide the eye the layout algorithm emphasises these communities and only positive functional connections with a link weight greater than 1.5 are shown with opacity reflecting the strength of interaction

To derive further mechanistic insight into the reasons for the selective patterns of functional interaction (Fig.7a, b), the overlap between the functional connectome and protein interactions was investigated. A focused Wnt Protein Interaction Network (PIN) was constructed based on gene lists of ‘Wnt regulators’ from four screens. These comprised an siRNA screen in the 7df3 cells (submitted manuscript), two siRNA screens in colon cancer [11, 12] and human homologues of the modulators identified in this report (Additional file 8: Table S5). These functionally identified regulators were used to interrogate the STRING protein interaction database [31] (experimental data only) to produce a protein interaction network comprising 699 nodes connected by 1846 links (grey lines). Community detection identified 20 different candidate protein complexes from the set of Wnt modulators (Louvain method for modularity maximization [32]; Fig. 7c, (Additional file 7: Table S3, Additional file 9: Figure S4, Additional file 10: Table S6). 16 of the functional connectome nodes identified in this study were present in the PIN, distributed amongst 8 complexes.

A prospective analysis suggested that there were more links between communities (candidate protein complexes) than within the same communities than would be expected by chance. This analysis compared the PIN community membership of the connectome’s links to shuffled versions of the connectome, (Additional file 11: Figure S5). Across the range of available thresholds the Z-score comparison of the actual connectome to the randomized versions was always positive (Additional file 11: Figure S5). This increase in links between communities supports the idea that functional interactions preferentially involved components of different protein complexes.

## Conclusions

In this report we identified 45 inducers and 96 inhibitors of TCF-dependent transcription using a cDNA expression screen. Known and novel regulators of Wnt signaling were identified. These included Prune, which is amplified in breast cancer [33] and was shown here to be required for the stabilisation of active β-catenin in MDA-MB231 breast cancer cells. Both inducers and inhibitors altered the absolute level of TCF-dependent transcription. The importance of the level of Wnt pathway activity has been highlighted by a study correlating discrete thresholds of activation with distinct developmental and oncogenic outcomes [34]. The overexpression (or loss of expression) of these regulators in cancer may modulate the absolute level of Wnt signaling such that levels that are ‘just right’ for discrete steps in oncogenic progression [35].

The conditional activation of TCF-dependent transcription with ∆NLRP prompted an analysis of cooperativity between multiple Wnt regulators. 466 of a possible 1170 pairwise combinations functionally interacted either positively or negatively. A key feature of the functional connectome was the non-overlapping patterns of functional connectivity (Fig. 7b). The mutually exclusive interaction patterns argue that non-overlapping cDNA pairs function via distinct underlying mechanisms. The pattern of interactions observed was termed a ‘functional connectome’ and can be displayed as a network graph (Fig. 4). Previous studies of Wnt regulators have identified enhancers and repressors of single-gene phenotypes such as APC-dependent tumorigenesis [36], but have not examined multi-gene interactions. Analogous pairwise-interaction studies have been carried out in yeast and Drosophila cells although synthetic lethality was a primary readout [37].

The functional connectome was derived from interactions that were identified in one reporter cell line. Nonetheless, many positive and negative interactions were also observed in the colon cancer cell line HCT116 suggesting that interactions and the lack of interaction may be conserved. Furthermore, functional interactions were restricted to less than 50 % of all potential interactions, suggesting that the network preserves context-dependence that may characterise the diverse array of cellular systems in which Wnt signaling functions. The functional connectome may then best be used as a map of potential interactions that can be used to guide studies based on the expression of network nodes.

### Highly connected network nodes and the ‘core pathway’

An observation from siRNA screens for novel Wnt signaling regulators was that ‘core’ pathway components were selectively enriched in the small subset of genes that were identified in more than one cell line. The functional connectome can help explain how proteins come to be regarded as ‘core’ components [1]. The structure of the functional connectome would suggest that a functional interaction between two gene products (A and B) would be observed following gene product ‘A’ depletion or overexpression only if its interacting partner ‘B’ were present/expressed. The degree of connectivity of the node would be critical. For example, expression of the transcriptional repressor SNAI2, as a relatively poorly connected node, might only modulate TCF dependent transcription in a cellular background in which one of its cooperating partners was expressed. By contrast changes to CK1ε levels/activity, as a well-connected node, would have a higher probability of altering Wnt signaling due to the greater probability of its interacting partners being expressed. On a probabilistic basis, highly connected, ubiquitously-expressed nodes would be most likely to be identified in multiple cell contexts. It is therefore possible that the concept of a ‘core’ Wnt pathway, which integrates results from multiple model systems, represents a statistical sample of a subset of highly-connected Wnt signaling nodes.

### Functional interactions in tumour cells

Fourteen enhancers super-activated TCF dependent transcription when transfected alone into HCT116 colon cancer cells (Fig. 3a, Additional file 4: Table S3). HCT116 cells already have raised levels of TCF-dependent transcription due to an activated β-catenin allele and autocrine Wnt signaling [18]. The promiscuous functional connections observed in HCT116 cells suggest that levels of TCF-dependent transcription may be more easily modulated once the pathway has initially been activated. Once the pathway has been activated by an initial mutation, the availability of many additional mechanisms for further pathway modulation may help melanomas, colon and liver cancers maintain ‘just right’ levels of Wnt signaling that have been proposed to be important for tumour formation [34, 35].

### Integrating functional and protein interaction networks

While a detailed mechanistic analysis of the interaction patterns observed is beyond the scope of this study, a rationale for functional connectivity was suggested when overlaps with protein interaction networks were explored (Fig. 7c, Additional file 11: Figure S5). Our analysis supported the hypothesis that functional connections preferentially linked proteins between discrete protein communities rather than proteins within a community [38] (Fig. 7, Additional file 9: Figure S4). As protein communities have been related to multiprotein complexes, and therefore to distinct molecular processes, this supports the idea that functional connections are more likely through alterations to distinct processes.

Protein overexpression can activate or inhibit the function of multiprotein complexes by titrating interacting components. The functional connectome may therefore represent a map of molecular processes that cooperate to regulate TCF-dependent transcription. Although nodes may not be physiological targets, they may nonetheless highlight molecular processes/protein complexes that synergise to generate graded levels of TCF-dependent transcription [34]. Functional connection patterns may also be helpful as a guide to potential mechanisms of action. For example, nodes with identical patterns may act through similar mechanisms. Likewise, similar patterns of activity of therapeutic inhibitors may be used to infer similar mechanisms of action. In the longer-term, the generation of larger networks and additional quantitative readouts should allow a greater level of prediction of context-specific signaling including the multiple novel genetic interactions observed in tumorigenesis [39].

### Therapeutic targeting of Wnt signaling

The list of novel Wnt pathway regulators identified by this and other screening approaches offer a range of potential targets for therapeutic intervention. Both Prune and HDGF are overexpressed in breast and colon cancers [30, 33, 40]. The phosphodiesterase activity of Prune may be well suited to the development of small molecule inhibitors, whilst antibodies to HDGF may be useful in the treatment of colorectal and liver cancer where it has been shown to act as an extracellular mitogen [30, 41]. H-ras and K-ras are primarily known for their oncogenic activation of MAP kinase signaling. However their identification here as Wnt enhancers is consistent with studies showing that MAP kinase signaling can modulate Wnt/β-catenin signaling and may be essential for the expression of Wnt target genes and tumour progression in colorectal cancer [42, 43].

The genetic uniqueness of individual cancers with respect to Wnt/TCF-dependent transcription signaling creates a dilemma for efforts to therapeutically target the pathway. Severe ‘off-target’ effects are predicted if ‘core’ pathway components are targeted given the homeostatic requirement for Wnt signaling in multiple tissues [44, 45]. The patterns of cooperativity identified in the functional connectome might be used to suggest combinations of inhibitors that enhance Wnt-specific phenotypic outputs whilst minimizing off-target toxicities [46].

## Methods

### Cell culture

All cells were cultured at 37 °C and 5 % CO_2_ in DMEM supplemented with 10 % heat inactivated Fetal Calf Serum, 50 units/ml Penicillin, 50 μg/ml Streptomycin and 0.5 % L-Glutamine 50 mg/ml (all from Gibco). 7df3 cells were maintained under constant antibiotic selection using 200 μg/ml hygromycin (Invitrogen) and 3 μg/ml blasticidin (Invitrogen). The identity of the SW480 and HCT116 lines was confirmed by DNA fingerprinting.

### DNA transfections and luminescence assays

Cells were transfected in 96-well format with 100 ng of plasmid DNA comprising CMV-driven cDNAs from the *Xenopus Tropicalis* library, the normalization control plasmid CMV-lacZ or the ‘filler’ plasmid pCDNA3.1 that also contains a CMV promoter. Transfection was performed using the Transfectin (BioRad) reagent according to manufacturers instructions. Assays were performed 48 h post transfection. Luciferase and galactosidase assays were performed using the Bright-Glo or Beta-Glo systems (Promega) respectively according to manufacturers instructions and assayed using a FluoStar Optima plate reader (BMG Labtech). β-catenin antibodies were used at 1/1000 dilution and were from BD Transduction Labs (Clone 14) and Merck Millipore (anti-ABC b-catenin clone 8E7). The anti-Flag (clone M2) antibody was from Sigma.

### Hit selection

Mean reporter activity was normalised as both a fold of the plate mean, and as the number of standard deviations away from the plate mean. The 100 most activating and 100 most inhibiting pools from each analysis were selected for deconvolution. To identify the responsible cDNA(s) within each well, each plasmid was co-transfected with ΔNLRP in triplicate. Hits that were significantly different from 36 ΔNLRP control wells (12 wells/96 well plate) were identified using a student’s two-tailed *t*-test (*p* < 0.01).

### Network studies

To identify and investigate synergistic interactions of co-expressed genes on the integrated β-catenin/TCF-reporter present within the 7df3 cells, we adopted a methodology based on that of Mani et al. for enumeration of genetic interaction [47]. Our observable output was a set of luminescence values indicating reporter activity. Luciferase values from individual (A or B) and pairwise (A co-transfected with B) cDNA combination in 7df3 cells (3–6 replicates) were first normalised to CMV-lacZ reporter levels and compared to background reporter activity from cells transfected with the pCDNA3.1 plasmid (*n* = 6–12 per 96-well plate).

The sets of values under scrutiny when evaluating the synergistic interaction between cDNAs A and B comprise the data obtained when A is expressed in isolation, when B is expressed in isolation and when both A and B are co-expressed. Additionally, we also recorded a background (control) scenario where no expression plasmid was expressed (other than the co-tranfected CMV-lacZ and ‘filler’ pCDNA3.1 control). We first computed the mean luminescences μA, μB and μA, B for each cDNA expressed in isolation and the pairwise co-expression (3–6 replicates). The datasets for each individual and the pairwise luminescences were compared to the control luciferase reporter activity. Those datasets that were not statistically significantly different (Kolmogorov-Smirnov (K-S) test; *p* < 0.01) including those with means higher than background were set to the background level. Datasets significantly different from background but with means lower than background were also set to the background level. This prevented the propagation of anomalies deriving from low-level fluctuations in reporter activity.

A network link weight (strength) for the synergistic interaction between nodes (cDNAs) A and B when co-expressed was then written:

$$ {W}_{a,b}= \log \left(\frac{\mu_{a,b}}{ \max \left({\mu}_a,{\mu}_b\right)}\right) $$

This enumeration of interaction has a number of salient properties. It prescribes positive link weights for synergistic interactions and negative weights for antagonistic interactions. A synergistic interaction is one in which pairwise co-expressed activity was greater than both individuals (i.e. the presence of one of the cDNAs enhanced the activity of the other). If only one of the pair activated when individually expressed, any increase above this would constitute a positive interaction. Similarly, if neither cDNA led to an increase in reporter activity above background levels when individually expressed, any reporter activity above background levels upon co-expression would constitute a synergistic interaction.

A negative link weight was prescribed for suppressive interactions in which pairwise activity is less than either one of the individuals. Consequently, if two cDNAs were co-expressed and either (or both) was capable of inducing reporter activity individually and the pairwise activity was lower than the maximum individual level, a negative link weight was assigned. The use of the logarithm ensured that the magnitudes of the multiplicative interactions are consistent between positive and negative synergies and that where no enhancement or suppression was observed; a zero edge weight was assigned (see Additional file 12: Supplementary Methods for details of mathematical analyses and model selection).

### Protein and functional interaction networks

A protein interaction network was generated from the list of Wnt-regulators (Additional file 8: Table S5) and first-degree protein interaction nodes as derived from the STRING database (see main text for details). Community detection algorithms were used to partition protein interaction network members (PIN) into groups of nodes (communities). See Additional file 12: Supplementary methods for further details. The subset of the functional connectome that had proteins in common with the PIN comprised 16.

Each functional connectome link was either between nodes in different PIN communities or lay within a community. To compare the number of connectome links that lay within and between PIN communities, the experimental connectome links were repeatedly randomized amongst the connectome nodes, in a fashion that preserved the degree of individual nodes (Maslov Sneppen method [20]. From this, a distribution of the number of inter-community links was created that was representative of a randomly selected network with the same degree sequence. The observed number of inter-community links in the original connectome was compared to this distribution via a z-score.

We further extended this comparison by considering only the strongest connectome links. We chose a threshold, τ, and retained only those connectome links with a weight greater than this threshold before repeating the randomization procedure.

### Analysis of cDNA inhibitors in cancer cell lines

Candidate inhibitor cDNAs were co-transfected with the TOPflash luciferase reporter in triplicate and were normalised to FOPflash (A control vector corresponding to TOPflash but instead containing scrambled TCF binding sites [2, 3]. A student’s two-tailed t-test and a Benjamini-Hochberg corrected significance cut-off of 0.01 was used to identify cDNAs capable of inhibiting TCF-dependent transcription driven by β-catenin mutation in HCT116 cells or by APC deletion (SW480).

### Functional analysis in *Xenopus laevis*

*X. laevis* embryos were obtained, cultured and injected as described [1, 3–5]. Experiments were carried out with relevant UK animal licence and ethical approvals. Capped mRNA for injection was prepared from the cDNA using the mMessage mMachine kit (Ambion), and purified using illustra ProbeQuant G-50 Micro Columns (GE Healthcare). RNA was purified and RT-PCR was performed as described [4, 6].

## Additional files

Additional file 1: Figure S1. Results of the primary screen. (EPS 2274 kb) Additional file 2: Table S1. List of all cDNAs that modulated TCF dependent transcription. (PDF 31 kb) Additional fie 3: Table S2. Summary of *Xenopus* data. (PDF 24 kb) Additional file 4: Table S3. Assessment of ‘Activator’ and ‘Inhibitor’ cDNA function in colon cancer cells. (PDF 52 kb) Additional file 5: Table S4. Separation of ‘enhancers’ and ‘activators’ based on differences from background. (PDF 48 kb) Additional file 6: Figure S2. Network nodes ordered by degree. (EPS 400 kb) Additional file 7: Figure S3. h-Prune synergises with super-activators. (EPS 437 kb) Additional file 8: Table S5. Wnt Regulator List. (PDF 184 kb) Additional file 9: Figure S4. PINs. (EPS 1226 kb) Additional file 10: Table S6. Gene and PIN community identifiers. (XLS 34 kb) Additional file 11: Figure S5. Functional connections are biased towards links between PIN communities. (EPS 249 kb) Additional file 12:
**Supplementary methods.** (DOC 213 kb)

## Abbreviations

ΔNLRP: a constitutively active form of the Wnt LRP6 co-receptor
PIN: Protein Interaction Network
TNBC: triple negative breast cancer
