# Supplementary material for: A functional connectome: regulation of Wnt/TCF-dependent transcription by pairs of pathway activators
Source: Mol Cancer. 2015 Dec 8;14:206. doi: 10.1186/s12943-015-0475-1 (PMC4672529; doi:10.1186/s12943-015-0475-1)
Supplement: Additional file 2: Table S1. — List of all cDNAs that modulated TCF dependent transcription. (PDF 31 kb) [file 12943_2015_475_MOESM2_ESM.pdf]

ACTIVATORS		
BLAST homologue gene name	Fold of deltaNLRP	p-value vs deltaNLRP
PRUNE2	12.73	2.06E-03
HRAS	28.49	5.48E-29
xCG8359-PA	2.47	1.87E-04
CSNK1E	6.05	5.15E-15
SLC12AB	5.72	1.11E-13
SOX21	11.36	2.06E-24
EMX2	3.88	5.70E-08
RBM5	3.03	1.64E-06
Myb protein P42POP	3.22	3.97E-06
GSC	3.80	1.63E-12
SNAI2	2.13	5.26E-04
IRX3	4.18	4.27E-08
HMGB1	16.06	3.73E-11
MNBH	1.81	8.24E-03
xNRAS	35.64	6.60E-22
HDGF	24.15	4.11E-30
ZFAND6	6.88	1.91E-15
HMGB3	5.82	1.04E-12
FOXP1	3.54	9.42E-11
CTSO	9.04	2.74E-12
UBE2E3	2.29	1.67E-04
WHSC1	3.54	4.77E-10
DVL2	3.94	5.13E-09
HMX2	8.43	2.22E-25
NKX6-2	5.28	4.38E-14
xZNF317	2.50	2.64E-06
xZNF300	1.88	2.82E-03
xZNF616	2.95	5.87E-08
xSON	2.33	4.24E-05
CSNK1D	5.15	8.01E-14
TPX2	1.99	8.77E-04
EMX1	2.50	2.63E-06
MAP3K7IP2	1.91	7.00E-03
FBL	2.79	7.62E-06
xZNF33A	2.42	2.92E-05
MIDN	2.07	9.05E-04
TGIF1	2.71	1.11E-09
RABEPK	2.49	3.86E-08
MESPA	3.20	7.78E-07
P2RY2	3.12	1.94E-11
WDR5	5.05	1.41E-11
Novel Zinc Finger	4.50	5.13E-16
CCDC18	2.63	4.11E-06
xKRAS2	8.52	1.80E-07
NUCKS1	3.11	1.32E-05

Supp Table 1

INHIBITORS		
BLAST homologue gene name	Fold of deltaNLRP	p-value vs. deltaNLRP
HES-1	0.01	1.32E-04
GPR107	0.11	5.12E-04
FOXA2	0.09	3.76E-04
HIST1H2AM	0.09	9.55E-05
RHOA	0.20	1.70E-03
HTPAP	0.16	9.33E-04
SERINC3	0.14	7.22E-04
CHCHD4	0.03	1.75E-04
EBAG9	0.11	4.90E-04
CASP10	0.21	1.67E-03
ARL8A	0.33	7.15E-03
CDC25A	0.15	8.02E-04
AXIN2	0.02	1.57E-04
xCHMP1	0.36	8.71E-03
CDC42BPB	0.06	2.68E-04
MRCKB	0.12	5.81E-04
TSPAN3	0.06	6.46E-05
PHB	0.07	2.73E-04
RPN1	0.00	1.16E-04
WDR82	0.27	3.52E-03
MGA3	0.04	1.87E-04
xTMEM150	0.15	7.93E-04
TMEM165	0.06	2.48E-04
STX5	0.08	3.34E-04
xSLC27A6	0.17	1.18E-03
PEX11B	0.21	1.80E-03
SCAP	0.25	2.89E-03
TMEM59	0.10	4.07E-04
FUBP1	0.07	2.93E-04
HGS	0.24	2.22E-03
No hits	0.16	9.54E-04
FAM57A	0.03	1.78E-04
T - Brachyury protein	0.16	9.55E-04
THOC4	0.01	1.34E-04
TBPL1	0.15	8.47E-04
Novel Protein	0.09	3.94E-04
SCOTIN	0.10	4.54E-04
CLCN5	0.26	3.45E-03
Novel protein similar to X-epilectin	0.06	6.31E-05
xCG6282-PA	0.01	1.29E-04
HSPA13	0.03	1.81E-04
POFUT1	0.00	1.16E-04
Novel 7 transmembrane receptor	0.09	3.61E-04
TMEM101	0.00	1.16E-04
xUGT3A1	0.09	3.89E-04
SOX7	0.00	1.16E-04
DLX2	0.02	1.55E-04
SRRP35	0.05	2.13E-04
xFUCOLECTIN	0.24	2.18E-03
HIST2H2AB	0.20	1.50E-03
CHSY1	0.14	7.17E-04
ATX	0.03	1.79E-04
RUNX2	0.08	3.56E-04
CFHR1	0.00	1.16E-04
WIPF2	0.17	1.14E-03
CANT1	0.07	2.80E-04
SOX17	0.05	1.94E-03
xPRS1	0.24	3.07E-03
EFEMP2	0.05	2.23E-04
TSKU	0.35	8.04E-03
UPK1	0.05	1.89E-03
ALCAM	0.06	2.59E-04
PRPF3	0.26	3.07E-03
RPS3	0.21	1.62E-03
TFAP2B	0.21	1.67E-03
RBM24	0.28	3.74E-03
BAT3	0.36	9.37E-03
NOL12	0.14	6.87E-04
Arachidonate 5-lipoxygenase variant	0.26	2.99E-03
CCDC18	0.24	2.36E-03
xMGC84823	0.15	7.53E-04
LH3	0.18	1.12E-03
TSC1	0.28	3.75E-03
IDH2	0.20	1.55E-03
STX10	0.03	1.62E-04
xTMEM46	0.12	1.40E-04
xWS1	0.11	1.24E-04
SERINC5	0.13	5.11E-04
SFRS3	0.54	6.52E-03
RXR-beta	0.28	1.23E-03
CACNA2D2	0.08	5.40E-03
DLX3	0.07	7.04E-05
SLC35A3	0.12	7.07E-03
POU5F1	0.07	4.53E-03
DERL1	0.13	8.01E-03
ZNT6	0.24	9.64E-04
FOXI1	0.23	2.99E-04
xMGC154907	0.52	3.10E-05
YTHDF2	0.36	1.03E-05
LACI	0.18	3.15E-06
ST3GAL2	0.18	1.71E-04
CDC42EP2	0.15	9.11E-03
MEX3C	0.30	8.28E-04
OTX2	0.42	1.30E-03
LYSMD2	0.06	2.33E-04
ZNF395	0.35	7.12E-03
