# Supplementary material for: A functional connectome: regulation of Wnt/TCF-dependent transcription by pairs of pathway activators
Source: Mol Cancer. 2015 Dec 8;14:206. doi: 10.1186/s12943-015-0475-1 (PMC4672529; doi:10.1186/s12943-015-0475-1)
Supplement: Additional fie 3: Table S2. — Summary of Xenopus data. (PDF 24 kb) [file 12943_2015_475_MOESM3_ESM.pdf]

Supp Table 2

Gene	Fold of deltaNLRP	Axis Duplication Assay		Target gene activation	
		n	Phenotype	Siamois	Xnr3
PRUNE2	12.73	20	3 partial axis duplication	+	NT
HMX2	6.57	21	3 complete axis duplication<br>1 partial axis duplication<br>17 anteriorised embryos	++	++
HMGB3	6.12	26	4 complete axis duplication<br>4 anteriorised embryos	++	++
HRAS	22.13	34	2 complete axis duplication<br>17 anteriorised embryos	NT	NT
EMX2	3.88	35	6 complete axis duplication<br>2 partial axis duplication<br>7 anteriorised embryos	++	++
HMGB1	16.06	18	1 complete axis duplication<br>4 partial axis duplication	++	++
HDGF	24.15	20	3 complete axis duplication<br>3 partial axis duplication	++	++
xZNF616	2.95	8	3 complete axis duplication	NT	NT
MESPA	3.2	40	2 partial axis duplication	NT	NT
KRAS2	8.52	23	2 partial axis duplication	-	NT
