# Supplementary material for: A functional connectome: regulation of Wnt/TCF-dependent transcription by pairs of pathway activators
Source: Mol Cancer. 2015 Dec 8;14:206. doi: 10.1186/s12943-015-0475-1 (PMC4672529; doi:10.1186/s12943-015-0475-1)
Supplement: Additional file 4: Table S3. — Assessment of ‘Activator’ and ‘Inhibitor’ cDNA function in colon cancer cells. (PDF 52 kb) [file 12943_2015_475_MOESM4_ESM.pdf]

Supp Table 3

ACTIVATORS	
Gene	HCT 116 Fold of TOPflash
PRUNE2	2.84
HRAS	1.28
xCG8359-PA	0.96
CSNK1E	3.53
SLC12A8	1.71
SOX21	1.89
EMX2	3.31
RBM5	1.16
P42POP	1.60
GSC	2.90
SNAI2	0.95
IRX3	4.87
HMGB1	1.16
MNBH	0.81
xNRAS	0.84
HDGF	2.10
ZFAND6	0.86
HMGB3	1.12
FOXP1	4.23
CTSO	1.07
UBE2E3	0.92
WHSC1	1.16
DVL2	1.47
HMX2	2.41
NKX6-2	2.42
xZNF317	1.11
xZNF300	1.37
xZNF616	1.13
xSON	0.98
CSNK1D	3.18
TPX2	0.92
EMX1	4.22
MAP3K7IP2	1.00
FBL	0.60
xZNF33A	1.10
MIDN	1.06
TGIF1	1.59
RABEPK	1.24
MESPA	0.61
P2RY2	0.74
WDR5	0.93
Novel Zinc Finger	1.37
CCDC18	1.01
xKRAS2	0.87
NUCKS1	0.65

INHIBITORS		
Gene	HCT 116 Fold of TOPflash	SW480 Fold of TOPflash
HES-1	1.12	0.76
GPR107	0.49	0.65
FOXA2	0.52	0.37
HIST1H2AM	0.41	0.65
RHOA	0.74	0.67
HTPAP	0.64	0.72
SERINC3	0.54	0.77
CHCHD4	0.71	0.48
EBAG9	0.80	0.76
CASP10	1.02	0.46
ARL8A	0.95	0.64
CDC25A	1.15	0.91
AXIN2	0.52	0.47
xCHMP1	0.74	0.96
CDC42BPB	1.08	0.60
MRCKB	0.45	0.61
TSPAN3	0.76	0.66
PHB	0.80	0.50
RPN1	1.15	0.49
WDR82	0.77	0.59
MGA3	0.72	0.52
xTMEM150	1.31	0.45
TMEM165	1.20	0.72
STX5	1.20	0.85
xSLC27A6	0.92	0.72
PEX11B	0.40	1.03
SCAP	1.03	0.62
TMEM59	0.99	0.63
FUBP1	1.38	1.20
HGS	1.43	0.69
No hits	1.17	0.62
FAM57A	0.95	0.77
T - Brachyury protein	1.24	0.51
THOC4	1.49	1.38
TBPL1	0.24	0.45
Novel Protein	0.87	0.92
SCOTIN	0.91	0.66
CLCN5	0.99	0.77
Novel protein similar to X-epilectin	0.65	0.42
xCG6282-PA	0.82	0.70
HSPA13	1.22	0.49
POFUT1	1.17	0.53
Novel 7 transmembrane receptor	0.79	0.73
TMEM101	0.93	0.75
xUGT3A1	1.14	0.61
SOX7	1.11	0.87
DLX2	1.02	0.80
SRRP35	1.56	1.16
xFUCOLECTIN	1.25	0.75
HIST2H2AB	0.81	0.60
CHSY1	1.56	0.70
ATX	0.84	0.91
RUNX2	0.93	0.60
CFHR1	1.38	0.54
WIPF2	1.50	0.73
CANT1	1.61	0.38
SOX17	1.10	0.58
xPRS1	0.94	1.00
EFEMP2	0.72	0.56
TSKU	1.32	0.97
UPK1	0.67	0.58
ALCAM	1.43	0.50
PRPF3	1.16	0.67
RPS3	0.86	0.56
TFAP2B	0.87	0.70
RBM24	3.64	0.95
BAT3	1.49	0.72
NOL12	0.91	1.02
Arachidonate 5-lipoxygenase variant	1.14	0.72
CCDC18	0.69	0.65
xMGC84823	0.89	0.63
LH3	0.70	0.42
TSC1	0.87	0.57
IDH2	0.44	0.31
STX10	1.05	0.42
xTMEM46	0.39	0.75
xWS1	0.51	0.55
SERINC5	0.78	0.30
SFRS3	0.45	0.50
RXR-beta	0.27	0.34
CACNA2D2	0.57	0.56
DLX3	1.22	0.29
SLC35A3	0.15	0.68
POU5F1	0.76	0.37
DERL1	1.12	0.49
ZNT6	0.67	0.48
FOXI1	1.45	0.41
xMGC154907	1.03	0.33
YTHDF2	0.70	0.40
LACI	0.77	0.44
ST3GAL2	0.79	0.37
CDC42EP2	1.43	0.62
MEX3C	1.22	0.44
OTX2	1.15	0.46
LYSMD2	1.54	0.72
ZNF395	0.72	0.34
