# Supplementary material for: A functional connectome: regulation of Wnt/TCF-dependent transcription by pairs of pathway activators
Source: Mol Cancer. 2015 Dec 8;14:206. doi: 10.1186/s12943-015-0475-1 (PMC4672529; doi:10.1186/s12943-015-0475-1)
Supplement: Additional file 5: Table S4. — Separation of ‘enhancers’ and ‘activators’ based on differences from background. (PDF 48 kb) [file 12943_2015_475_MOESM5_ESM.pdf]

Supp Table 4

cDNA	2 tail Kstest vs. Background pval	individual luminescence distribution mean
VP16-TCF	4.67E-34	0.93741
VP16-minTCF	1.35E-39	71.6452
AxinGID	1.35E-39	5.5356
deltaNbetaCAT	2.18E-21	0.82662
PRUNE2	1.17E-50	7.2291
HRAS	0.2527	0.082585
xCG8359-PA	0.040247	0.069695
CSNK1E	3.64E-16	0.40181
SLC12A8	0.17065	0.090698
SOX21	0.20693	0.10837
EMX2	0.31411	0.11068
RBM5	0.0027637	0.03988
Myb protein P42POP	0.00020805	0.032569
GSC	0.042257	0.054336
SNAI2	0.16538	0.079256
IRX3	0.12947	0.071123
HMGB1	0.092278	0.10919
MNBH	0.0052904	0.044614
xNRAS	0.37724	0.14001
HDGF	0.041071	0.17337
ZFAND6	0.60228	0.6814
HMGB3	0.36769	0.15892
FOXP1	0.061325	0.36794
CTSO	0.33106	0.19178
UBE2E3	0.74964	0.086688
WHSC1	0.3935	0.13748
DVL2	8.39E-47	4.0706
HMX2	0.40681	0.071941
NKX6-2	0.46803	0.13599
xZNF317	0.7913	0.11385
xZNF300	0.30946	0.15351
xZNF616	0.055061	0.070659
xSON	0.42899	0.13568
CSNK1D	0.0090535	0.16577
TPX2	0.14774	0.10531
EMX1	0.68849	0.13841
MAP3K7IP2	0.8541	0.089736
FBL	0.44298	0.069187
xZNF33A	0.40179	0.074135
MIDN	0.19543	0.12543
TGIF1	0.67447	0.10139
RABEPK	0.89054	0.094314
MESPA	4.19E-09	0.44644
P2RY2	0.74709	0.10727

WDR5	0.18635	0.095215
Novel Zinc Finger	0.17847	0.072922
CCDC18	0.12018	0.051403
xKRAS2	0.74709	0.12165
NUCKS1	0.38118	0.08982
