# Supplementary material for: A functional connectome: regulation of Wnt/TCF-dependent transcription by pairs of pathway activators
Source: Mol Cancer. 2015 Dec 8;14:206. doi: 10.1186/s12943-015-0475-1 (PMC4672529; doi:10.1186/s12943-015-0475-1)
Supplement: Additional file 8: Table S5. — Wnt Regulator List. (PDF 184 kb) [file 12943_2015_475_MOESM8_ESM.pdf]

Gene Symbol (Gene Name)	Ensembl Gene (ENSG)	7df3 cDNA Hit	7df3 esiRNA hits	DLD-1 siRNA Hit	HeLa siRNA Hit
A1BG	ENSG00000121410			1	
A2M	ENSG00000175899			1	
AAK1	ENSG00000115977			1	
ABHD12	ENSG00000141519			1	
ACAA1	ENSG00000060971			1	1
ACADSB	ENSG00000196177			1	
ACD	ENSG00000102977			1	
ACP2	ENSG00000023839|ENSG00000134575			1	
ACTL6A	ENSG00000136518			1	
ADAM15	ENSG00000143537			1	
ADAM21	ENSG00000099139|ENSG00000139985			1	
ADAM32	ENSG00000197140			1	
ADAMTSL5	ENSG00000185761			1	
ADAT3	ENSG00000213638			1	
ADK	ENSG00000075624			1	
AES	ENSG00000104964|ENSG00000130402			1	
HSPC103				1	
AFP	ENSG00000114739			1	
AGBL3	ENSG00000146856			1	
AGGF1	ENSG00000164252			1	
AGMAT	ENSG00000116771			1	
AK026675				1	
AK055252				1	
AK124784				1	
AK125430				1	
AK127029				1	
AK127085				1	
AK127100				1	
AK128824				1	
AKAP5	ENSG00000179841			1	
AKT1	ENSG00000142208|ENSG00000168594			1	

ALMS1	ENSG00000116127			1	
NBEAL1	ENSG00000144426		1	1	
AMFR	ENSG00000159461			1	
AMICA1	ENSG00000160593			1	
ANAPC2	ENSG00000176248			1	
ANAPC4	ENSG00000053900			1	
ANGEL1	ENSG00000013523|ENSG00000232119			1	
ANKRD13B	ENSG00000198720			1	
ANKRD24	ENSG00000089847			1	
ANKRD57	ENSG00000198142			1	
ANKRD6	ENSG00000135299			1	
AOF2	ENSG00000004487			1	
AP3B1	ENSG00000122884|ENSG00000132842			1	
APBA1	ENSG00000204305|ENSG00000206320|ENSG00000229058|ENSG0000			1	
APC	ENSG00000204310|ENSG00000206324|ENSG0000		1	1	1
APC2	ENSG00000115266			1	
APPBP1	ENSG00000159593		1	1	1
ARID1A	ENSG00000117713			1	
ARID1B	ENSG00000049618			1	
ARID2	ENSG00000189079			1	
ARID3A	ENSG00000116017|ENSG00000124469			1	
ARMET	ENSG00000249398			1	
ARVCF	ENSG00000099889			1	
ASCL2	ENSG00000179477|ENSG00000183734			1	
ASH2L	ENSG00000115956|ENSG00000129691			1	
ASPM	ENSG00000066279			1	
ATAD3A	ENSG00000197785			1	
ATF7IP	ENSG00000171681		1	1	1
ATP1A2	ENSG00000243480			1	
AURKB				1	
AXIN2	ENSG00000168646	1	1	1	1
AXL	ENSG00000167601			1	

AYTL1	ENSG00000087253	1	
BAG4	ENSG00000156735	1	
BAIAP2	ENSG00000175866|ENSG00000215695	1	
BANF1	ENSG00000175334	1	
BC028232		1	
BC031108		1	
BC069767		1	
BCL6	ENSG00000111701	1	
BCL6B	ENSG00000161940	1	
BCL9	ENSG00000116128	1	
BCR	ENSG00000130203	1	
BLID	ENSG00000221871	1	
BM924854		1	
BOP1	ENSG00000138347|ENSG00000170727	1	
BPIL2	ENSG00000184459	1	
BRCA1	ENSG00000177105	1	
BRD7	ENSG00000166164	1	
BTRC	ENSG00000114204	1	
BXDC5	ENSG00000117133	1	
C10orf72	ENSG00000165633	1	
C12orf12	ENSG00000197651	1	
C12orf39	ENSG00000134548	1	
C13orf31	ENSG00000179630	1	
C14orf65	ENSG00000205476	1	
C17orf76	ENSG00000181350	1	
IZUMO2	ENSG00000161652	1	
C19orf50	ENSG00000105700	1	
C1D	ENSG00000197223	1	
C1orf104	ENSG00000225855	1	
BANF2	ENSG00000125888	1	
C20orf195	ENSG00000125531	1	
C20orf29	ENSG00000125843	1	1

C20orf4	ENSG00000131043		1	
C20orf43	ENSG00000022277	1	1	
C21orf109			1	
C3orf67	ENSG00000163689		1	
C7orf27	ENSG00000106009		1	
C8orf57			1	
C9orf27	ENSG00000204148		1	
C9orf4	ENSG00000136805		1	
C9orf93	ENSG00000164989		1	
CACNA1A	ENSG00000152092		1	1
CAMK2B	ENSG00000067842		1	
CBY1	ENSG00000100211		1	
CCDC32	ENSG00000128891		1	
CCDC88	ENSG00000168071		1	
CCL16	ENSG00000161573		1	
CCNB1	ENSG00000134057		1	
CD247	ENSG00000198821|ENSG00000206285|ENSG00000226936|ENSG0000		1	
CD81	ENSG00000116752		1	
CDC2L1	ENSG00000008128		1	
CDC37	ENSG00000074317|ENSG00000105401		1	
CDC42	ENSG00000069399		1	
CDC42EP2	ENSG00000072133|ENSG00000149798	1	1	
CDC73	ENSG00000134371		1	
CDCA2	ENSG00000184661		1	
CDCA7L	ENSG00000164649		1	
CDK5	ENSG00000074582		1	
CDKN2A	ENSG00000147889		1	
CDKN3	ENSG00000100526		1	
CEACAM21	ENSG00000007129		1	
CEACAM5	ENSG00000105388		1	
CENPQ	ENSG00000031691		1	
CES3	ENSG00000172828		1	

CGA	ENSG00000169594		1	
CGB	ENSG00000104827|ENSG00000189052|ENSG00000213030		1	
CGN	ENSG00000143375		1	
CHAF1B	ENSG00000136834		1	
CHD1	ENSG00000137948|ENSG00000153922		1	
CHD8	ENSG00000100888		1	
CIDEA	ENSG00000156970|ENSG00000176194		1	
CISD1	ENSG00000122873		1	
CLCNKA	ENSG00000186510		1	
CLDND2	ENSG00000160318		1	
CNGA3	ENSG00000144191		1	
CNOT7	ENSG00000186074		1	
COL11A2	ENSG00000204248|ENSG00000230930		1	
COL20A1	ENSG00000101203		1	
CORIN	ENSG00000100003		1	
CORO2A	ENSG00000106789		1	
CREBBP	ENSG00000005339		1	
CRISP1	ENSG00000138107		1	
CRKRS	ENSG00000167258		1	
CRYGN	ENSG00000127377		1	
CSAG1	ENSG00000198930		1	
CSNK1A1	ENSG00000178363		1	
CSNK1E	ENSG00000213923		1	1
CSNK2A2	ENSG00000134072		1	
CSTF1	ENSG00000101138|ENSG00000142330		1	
CSTP1	ENSG00000103381		1	
CTBP1	ENSG00000159692		1	
CTBP2	ENSG00000116489		1	
CTNNB1	ENSG00000137752	1	1	1
CTNNBIP1	ENSG00000178585		1	
CTR9	ENSG00000163629|ENSG00000198730		1	
CTRC	ENSG00000139874		1	

CTSE	ENSG00000118412	1
CUEDC2	ENSG00000107874	1
CUTA	ENSG00000226492	1
CXXC4	ENSG00000168772	1
CXXC5	ENSG00000171604	1
CYP26B1	ENSG00000003137	1
CYP2F1	ENSG00000197446	1
DAAM1	ENSG00000100592	1
DACT1	ENSG00000165617	1
DACT3	ENSG00000197380	1
DCLK1	ENSG00000115138	1
DDX19B	ENSG00000164056	1
DDX27	ENSG00000124228	1
DDX39	ENSG00000123136	1
DDX54	ENSG00000123064	1
DFFB	ENSG00000169598	1
DGAT2L4	ENSG00000147160	1
DHH	ENSG00000139549	1
DHX40	ENSG00000108406	1
DIS3L	ENSG00000166938	1
DIXDC1	ENSG00000150764	1
DNAJA2	ENSG00000116745	1
DNAJC6	ENSG00000132155	1
DOCK4	ENSG00000128512	1
DPF2	ENSG00000133884|ENSG00000164136	1
DSC2	ENSG00000134755	1
DUX1		1
DYNC1LI1	ENSG00000144635	1
E4F1	ENSG00000167967	1
ECM2	ENSG00000106823	1
EDA	ENSG00000158813	1
EDEM3	ENSG00000116406	1

EED	ENSG00000074266	1
EEF1A2	ENSG00000173575	1
EGR1	ENSG00000101204	1
EHD2	ENSG00000024422|ENSG00000176040	1
EHMT2	ENSG00000206376	1
EIF2AK2	ENSG00000055332	1
EIF4A3	ENSG00000141543	1
EIF4E	ENSG00000151247	1
EIF4EBP3	ENSG00000243056	1
EML4	ENSG00000143924	1
ENPEP	ENSG00000138792	1
ENST00000222607	ENSG00000105889	1
ENST00000297801		1
ENST00000325394	ENSG00000181508	1
ENST00000327725		1
ENST00000380670		1
ENST00000382568		1
ENST00000390299	ENSG00000211653	1
ENST00000390331	ENSG00000211685	1
EP300	ENSG00000134873	1
EP400	ENSG00000183495	1
EPB41L3	ENSG00000163156	1
EPB41L4A	ENSG00000129595	1
EPHA4	ENSG00000116106	1
EPPK1		1
ERCC6	ENSG00000225830	1
ETHE1	ENSG00000105755|ENSG00000148483|ENSG00000184040	1
EXO1	ENSG00000187021	1
EXOSC10	ENSG00000205403	1
EXOSC6	ENSG00000223496	1
EXOSC9	ENSG00000162783	1
EZH2	ENSG00000106462	1

F2RL1	ENSG00000144191|ENSG00000164251		1	
FAM115A	ENSG00000198420		1	
FAM126A	ENSG00000122591		1	
FAM134A	ENSG00000144567		1	
FAM5C	ENSG00000162670		1	
FAM87B	ENSG00000177757		1	
FBXL12	ENSG00000127452		1	1
FBXL15	ENSG00000107872		1	
FBXO33	ENSG00000165355		1	
FBXO38	ENSG00000145868		1	
FBXW11	ENSG00000072803		1	1
FEN1	ENSG00000168496		1	
FEV	ENSG00000163497		1	
FLJ22662	ENSG00000121316		1	
CCDC150	ENSG00000144395		1	
HEATR7B2	ENSG00000171495		1	
FLJ40852	ENSG00000228775		1	
SHISA6	ENSG00000188803		1	
FNDC5	ENSG00000160097	1	1	
FOLR3	ENSG00000095794|ENSG00000110203		1	
FOS	ENSG00000170345		1	
FTSJ3	ENSG00000108592		1	
FUT10	ENSG00000172728		1	
FXYD5	ENSG00000089327		1	
FZD5	ENSG00000163251		1	
GABRE	ENSG00000102287		1	
GANC	ENSG00000214013		1	
GAS1	ENSG00000180447		1	
GCK	ENSG00000106633		1	
GCN5L2	ENSG00000108773		1	
GDA	ENSG00000119125		1	
GDI1	ENSG00000203879		1	

GFRA4	ENSG00000125861	1
GJA5	ENSG00000143140	1
GLB1L3	ENSG00000166105	1
GNB2L1	ENSG00000204628	1
GNL3L	ENSG00000130119	1
GPAM	ENSG00000119927	1
GPBAR1	ENSG00000179921	1
GPI	ENSG00000105220	1
GPR114	ENSG00000159618	1
GPR120	ENSG00000186188	1
GPR173	ENSG00000184194	1
GPR84	ENSG00000139572	1
GPX4	ENSG00000180096	1
GRK6	ENSG00000198055	1
GRM1	ENSG00000152822	1
GSK3A	ENSG00000105723	1
GSK3B	ENSG00000152592	1
GSS	ENSG00000172869	1
GUCY1A3	ENSG00000164116	1
HBP1	ENSG00000105856	1
HCCS	ENSG00000096696	1
HCFC1	ENSG00000172534	1
HCRTR1	ENSG00000168393	1
HDAC1	ENSG00000143507	1
HDAC2	ENSG00000144048	1
HDAC3	ENSG00000171720	1
HDAC8	ENSG00000147099	1
HDAC9	ENSG00000048052	1
HERC4	ENSG00000148634	1
HHATL	ENSG00000010282	1
HIAT1	ENSG00000156875	1
HIATL1	ENSG00000148110	1

HIPK2	ENSG00000137815		1
HIST1H2BM	ENSG00000196374		1
HLA-DOB	ENSG00000241106		1
HMGN2	ENSG00000198830		1
HNF1A	ENSG00000135100		1
HNF1B	ENSG00000108753		1
HNRPA1	ENSG00000124205|ENSG00000135486	1	1
HNRPCL1	ENSG00000179172		1
HNRPUL1	ENSG00000105323	1	1
HOXB3	ENSG00000120093		1
HSP90AA2	ENSG00000080824		1
HSPA4	ENSG00000170606		1
HSPA9	ENSG00000107105		1
HSPD1	ENSG00000144381		1
HTATIP	ENSG00000172977		1
IAPP	ENSG00000121351		1
ICK	ENSG00000112144		1
ICMT	ENSG00000116237|ENSG00000188234		1
IDE	ENSG00000119912		1
IFIT5	ENSG00000152778		1
IGHG3			1
IGLV3-21			1
IGLV4-3			1
IKZF1	ENSG00000185811		1
IL3RA	ENSG00000205186		1
INF2	ENSG00000203485		1
INOC1	ENSG00000128908		1
INTS1	ENSG00000164880		1
INTS6	ENSG00000162819		1
ITGA11	ENSG00000137809		1
ITPK1	ENSG00000100605		1
ITPR3	ENSG00000096433		1

JAK2	ENSG00000096968		1	
JPH3	ENSG00000154118		1	
JRK	ENSG00000234616		1	
SKP1A	ENSG00000113558	1	1	
KCNK13	ENSG00000152315		1	
KCNMB4	ENSG00000135643		1	
KHK	ENSG00000138030		1	
KIAA0090	ENSG00000127463		1	
KIAA0174	ENSG00000182149		1	
KIAA0319	ENSG00000137261		1	
ZC4H2	ENSG00000126970		1	
ERMN	ENSG00000136541	1	1	
G2E3	ENSG00000092140		1	
KIAA1549	ENSG00000122778		1	
KIAA2022	ENSG00000050030		1	
KIF15	ENSG00000163808		1	
KLF1	ENSG00000105610|ENSG00000124145		1	
KLHL12	ENSG00000117153		1	
KLRC3	ENSG00000114861|ENSG00000205810		1	
KRT1	ENSG00000167768		1	
KRT39	ENSG00000196859		1	
KRTAP12-3	ENSG00000205439		1	
KRTAP19-1	ENSG00000184351		1	
KRTAP9-2	ENSG00000239886		1	1
LACTB	ENSG00000103642		1	
LAGE3	ENSG00000171180		1	
LAMB1	ENSG00000091136		1	
LBX1	ENSG00000138136|ENSG00000162512		1	
LCN1	ENSG00000160349		1	
LEF1	ENSG00000138795	1	1	
LEFTY2	ENSG00000131446		1	
LEO1	ENSG00000166477		1	

LGALS1	ENSG00000100097			1	
LHFPL5	ENSG00000197753			1	
LLGL2	ENSG00000073350			1	
LRRN4CL	ENSG00000177363			1	
LRRC67	ENSG00000178125			1	
LOC338651				1	
LOC400464				1	
LOC400794	ENSG00000237463			1	
LONRF2	ENSG00000170500			1	
LRBA	ENSG00000083123			1	
LRRC15	ENSG00000172061			1	
LRRC44	ENSG00000162620			1	
LRRK1	ENSG00000154237			1	
LRRN2	ENSG00000166592			1	
LRRTM1	ENSG00000162951			1	
LTF	ENSG00000160211			1	
LUZP1	ENSG00000169641			1	
LYSMD2	ENSG00000140280	1		1	1
LYSMD3	ENSG00000176018			1	
LZIC	ENSG00000162441			1	
M6PR	ENSG00000003056|ENSG00000104064			1	1
MAG	ENSG00000105695|ENSG00000189064			1	
MAGEC3	ENSG00000165509			1	
MANSC1	ENSG00000111261		1	1	
MAP3K10	ENSG00000130758			1	
MAP3K7	ENSG00000135341			1	
TAB1	ENSG00000100324			1	
MAP3K8	ENSG00000107968			1	
MAPK13	ENSG00000156711			1	
MASP1	ENSG00000127241			1	
MASP2	ENSG00000009724			1	
MAST2	ENSG00000177182			1	

MAMSTR	ENSG00000176909	1	
MCM3AP	ENSG00000160294	1	
MDN1	ENSG00000112159	1	
MEX3D	ENSG00000181588	1	1
MGST3	ENSG00000131067	1	
MIF4GD	ENSG00000125457	1	
Mirn126		1	
MIRN99A		1	
MKNK1	ENSG00000079277	1	
MKRN1		1	
MKRN2	ENSG00000075975	1	
MLH3	ENSG00000119684|ENSG00000168724	1	
MLL	ENSG00000118058	1	
MLL2	ENSG00000167548	1	
MLLT6	ENSG00000108292	1	
MMP1	ENSG00000196611	1	
MMP16	ENSG00000065325|ENSG00000156103	1	
MMP19	ENSG00000123342	1	
MMP3	ENSG00000149968	1	
MON1A	ENSG00000164077	1	
MOXD1	ENSG00000079931	1	
MRPL49	ENSG00000149792	1	
MYLK	ENSG00000084207	1	
NAPEPLD	ENSG00000161048	1	
NARG2	ENSG00000128915	1	
NDUFB8	ENSG00000189060	1	
NDUFV1	ENSG00000167792	1	
NEK6	ENSG00000119408	1	
NEURL2	ENSG00000124257	1	
NF2	ENSG00000112727|ENSG00000124693|ENSG00000182572|ENSG0000	1	
NHS	ENSG00000116882	1	
NKX1-1	ENSG00000235608	1	

NKX3-1	ENSG00000167034|ENSG00000188536|ENSG00000206172	1	
NLGN3	ENSG00000196338	1	
NLK	ENSG00000087095	1	1
NOL3	ENSG00000140939	1	
NOS1	ENSG00000089250	1	
NOTCH4	ENSG00000140287	1	
NOX4	ENSG00000086991	1	
NPLOC4	ENSG00000182446	1	
NPTX2	ENSG00000106236	1	
NR2C2	ENSG00000177463	1	
NT5C2	ENSG00000076685	1	
NUCB1	ENSG00000104805	1	
NUDT10	ENSG00000122824	1	
NUP153	ENSG00000163918	1	
NUSAP1	ENSG00000137804	1	
NYX	ENSG00000188937	1	
OAZ1	ENSG00000104904	1	
OLIG1	ENSG00000184221	1	
OPA3	ENSG00000125741	1	
OPN1LW	ENSG00000163501	1	
OR6C2	ENSG00000179695	1	
OSBPL11	ENSG00000144909	1	
OSBPL3	ENSG00000070882	1	
OSGIN2	ENSG00000164823	1	
OSMR	ENSG00000145623	1	
OXA1L	ENSG00000155463	1	
PACSIN1	ENSG00000101883	1	
PAF1	ENSG00000006712	1	
PAG1	ENSG00000076641	1	
PAIP2	ENSG00000120727	1	
PAK3	ENSG00000077264	1	
PALB2	ENSG00000083093	1	

PALLD	ENSG00000198477		1	
PALM	ENSG00000099864		1	
PARP3	ENSG00000041880		1	
PAX7	ENSG00000009709		1	
PBRM1	ENSG00000163939		1	
PCDHGB1			1	1
PCK2	ENSG00000100889		1	
PCSK2	ENSG00000198353		1	
CDK17	ENSG00000059758		1	
PCYOX1L	ENSG00000145882		1	
PDZRN3	ENSG00000173163		1	
PELI1	ENSG00000197329		1	
PES1	ENSG00000188716		1	
PGD	ENSG00000160207		1	
PGM2	ENSG00000169299		1	
PGM3	ENSG00000044574		1	
PHACTR2	ENSG00000112419		1	
PHF5A	ENSG00000100410		1	
PHOX2B	ENSG00000109132		1	
PIK3C3	ENSG00000078142|ENSG00000179546		1	
PIM1	ENSG00000179097		1	
PIM3	ENSG00000198355		1	
PINK1	ENSG00000158828		1	
PITX2	ENSG00000164093	1	1	1
PKDREJ	ENSG00000130943		1	
PKM2	ENSG00000067225		1	
PLAGL2	ENSG00000126003		1	
PLEKHM1	ENSG00000160271|ENSG00000225190|ENSG00000233758		1	
PLGLA1	ENSG00000169659		1	
PLK1	ENSG00000166851		1	
PMPCA	ENSG00000165688		1	
PMS2L3	ENSG00000067064		1	

PNN	ENSG00000100941|ENSG00000119917		1	
PNPT1	ENSG00000138035		1	
POLD4	ENSG00000175482		1	
POLR2E	ENSG00000099817|ENSG00000171855		1	1
POLR2G	ENSG00000168002		1	
POLR2H	ENSG00000163882		1	
POMC	ENSG00000115138		1	1
PPAPDC1A	ENSG00000203805	1	1	
PPBPL2	ENSG00000119541		1	
PPEF1	ENSG00000086717		1	
PPIAL4	ENSG00000198161		1	
PPIB	ENSG00000166794		1	
PPIF	ENSG00000108179		1	
PPP1CC	ENSG00000186298		1	
PPP1R12B	ENSG00000077157		1	
PPP1R15A	ENSG00000087074		1	
PPP1R16B	ENSG00000056050		1	
PPP2R4	ENSG00000119383		1	
PRG2	ENSG00000186652		1	
PRICKLE2	ENSG00000163637		1	
PRICKLE4	ENSG00000124593		1	
PRMT7	ENSG00000103064		1	
PROCR	ENSG00000101000		1	
PRPF8	ENSG00000136546		1	
PRR14	ENSG00000156858		1	
PRSS23	ENSG00000080503		1	
PRSSL1	ENSG00000185198		1	
PRUNE	ENSG00000143363		1	
PRY	ENSG00000083444		1	
PSEN1	ENSG00000080815		1	
PSMB10	ENSG00000205220		1	
PSMB7	ENSG00000136930		1	

PUS1	ENSG00000177192	1
PEX2	ENSG00000164751	1
PYCARD	ENSG00000103490	1
PYDC1	ENSG00000169900	1
PYGM	ENSG00000068976	1
PYGO1	ENSG00000115295	1
QPRT	ENSG00000213185	1
RAD17	ENSG00000152942	1
RAD54L	ENSG00000085999	1
RARA	ENSG00000131759	1
RASGRP3	ENSG00000152689	1
RB1	ENSG00000139687	1
RBBP5	ENSG00000117222	1
DNAJC27	ENSG00000115137	1
RBP3	ENSG00000134256	1
RCOR1	ENSG00000089902	1
REEP1	ENSG00000068615	1
RENBP	ENSG00000169194	1
REPS2	ENSG00000169891	1
RFC3	ENSG00000133119	1
RGS16	ENSG00000174564	1
RHOBTB3	ENSG00000164292	1
RIMBP2	ENSG00000060709|ENSG00000172671	1
RLBP1	ENSG00000091181|ENSG00000140522	1
RNF7	ENSG00000114125	1
ROPN1L	ENSG00000145491	1
RORA	ENSG00000069667|ENSG00000245534	1
LONRF3	ENSG00000175556	1
RPL7L1	ENSG00000146223	1
RPRML	ENSG00000179673	1
RPS21	ENSG00000171858	1
RPS6KA1	ENSG00000117676	1

RPS6KA2	ENSG00000008083|ENSG00000071242	1	
RPS6KB1	ENSG00000108443	1	
RPUSD4	ENSG00000165526	1	
RRP9	ENSG00000114767	1	
RSRC1	ENSG00000174891	1	
RTF1	ENSG00000137815	1	
RUVBL1	ENSG00000185345	1	
RUVBL2	ENSG00000183207	1	
S100A1	ENSG00000160678	1	
SAA1	ENSG00000173432	1	
SAE1	ENSG00000142230	1	
SAE2	ENSG00000126261	1	
SBNO1	ENSG00000139697	1	
SCN2B	ENSG00000151657	1	
SCNN1D	ENSG00000162572|ENSG00000240403	1	
SERTAD3	ENSG00000167565	1	
SESN2	ENSG00000130766	1	
SETD7	ENSG00000145391	1	
SH2B3	ENSG00000111252|ENSG00000137275	1	
SIAH1	ENSG00000167613	1	
SIM2	ENSG00000058085|ENSG00000159263|ENSG00000248691|ENSG0000	1	1
SKIL	ENSG00000136603	1	
SLAMF8	ENSG00000158714	1	
SLC15A3	ENSG00000110446	1	
SLC17A4	ENSG00000135828	1	
SLC25A23	ENSG00000125648	1	
SLC25A37	ENSG00000147454	1	
SLC25A39	ENSG00000013306	1	
SLC26A4	ENSG00000091137|ENSG00000168453	1	
SLC4A5	ENSG00000188687	1	
SLC6A2	ENSG00000115850	1	
SLC7A1	ENSG00000111716	1	

SLC7A11	ENSG00000151012	1	
SMAD3	ENSG00000166949	1	
SMAD4	ENSG00000141646	1	
SMARCA1	ENSG00000106689	1	
SMARCA2	ENSG00000107187	1	
SMARCA4	ENSG00000113594|ENSG00000127616	1	
SMARCA5	ENSG00000170920	1	
SMARCB1	ENSG00000099956	1	
SMARCC1	ENSG00000173473	1	
SMARCC2	ENSG00000139613|ENSG00000174405	1	
SMARCD1	ENSG00000104974	1	1
SMARCD2	ENSG00000108604	1	
SMARCD3	ENSG00000082014	1	
KRT222P		1	1
SNCB	ENSG00000074317	1	
SNX9	ENSG00000130340	1	
SOCS3	ENSG00000184557	1	
SOD1	ENSG00000163431	1	
SORT1	ENSG00000135750	1	
SOX1	ENSG00000113441	1	
SPATA20	ENSG00000006282	1	
SQSTM1	ENSG00000067057	1	
SRGAP1	ENSG00000196935	1	
SRMS	ENSG00000125508	1	
SSPN	ENSG00000123096|ENSG00000188937	1	
SSR1	ENSG00000124783	1	
ST8SIA1	ENSG00000111728	1	
STARD6	ENSG00000174448	1	
STK11	ENSG00000118046	1	
STK17A	ENSG00000164543	1	
STK19	ENSG00000204344|ENSG00000226257	1	
STK40	ENSG00000196182	1	

STX1B	ENSG00000099365		1	
SYT16	ENSG00000139973		1	
SYT8	ENSG00000149043		1	
TACR2	ENSG00000011566		1	
TACSTD2	ENSG00000204681|ENSG00000206466|ENSG00000206511|ENSG0000		1	
TAF1A	ENSG00000130943|ENSG00000143498		1	
tAKR			1	
TAX1BP3	ENSG00000213977		1	
TAZ	ENSG00000127241		1	
TCF3	ENSG00000071564		1	
TCF4	ENSG00000197971		1	
TCF7	ENSG00000081059	1	1	1
TCF7L1	ENSG00000152284		1	
TCF7L2	ENSG00000148737		1	1
TDP1	ENSG00000042088		1	
TF	ENSG00000091513		1	
TIAM2	ENSG00000168955		1	1
TIMM17A	ENSG00000134375|ENSG00000171863		1	
TIMP2	ENSG00000035862		1	
TK2	ENSG00000166548		1	
TLE1	ENSG00000196781	1	1	
TLE2	ENSG00000065717		1	
TLE3	ENSG00000140332|ENSG00000183214|ENSG00000204520|ENSG0000		1	
TLE6	ENSG00000104953		1	
TMEM139	ENSG00000178826		1	
TMEM2	ENSG00000135048		1	
TMEM55A	ENSG00000155099		1	
TMEM55B	ENSG00000165782		1	
TMPRSS11D	ENSG00000184304		1	
TNFRSF13C	ENSG00000159958		1	
TNFSF13B	ENSG00000102524		1	
TNIP2	ENSG00000168884		1	

TRAK1	ENSG00000182606		1	
TRAV20			1	
TRIM27	ENSG00000137496	1	1	
TRIM28	ENSG00000130726		1	
TRIM6	ENSG00000242885		1	1
TRIP10	ENSG00000011485		1	
TRRAP	ENSG00000196367		1	
TSPAN15	ENSG00000099282		1	
TTC18	ENSG00000156042		1	
TTC21A	ENSG00000168026		1	
TTLL5	ENSG00000119685|ENSG00000174326		1	
TTTY8			1	
TUBB3	ENSG00000163902|ENSG00000198211		1	
TXLNA	ENSG00000084652		1	
TXNRD1	ENSG00000198431		1	
UBE2R2	ENSG00000107341		1	
UBE2Z	ENSG00000159202		1	
UBL5	ENSG00000198258		1	
UBR4	ENSG00000083535|ENSG00000127481		1	
UBXD2	ENSG00000144224		1	
UBXD8	ENSG00000113194		1	
UGT2B7	ENSG00000171234		1	
SUN1	ENSG00000164828		1	
UQCR	ENSG00000127540		1	
USP48	ENSG00000090686		1	
USP53	ENSG00000145390		1	
UTP18	ENSG00000011260		1	
VASH1	ENSG00000071246		1	
VAV1	ENSG00000141968		1	
VAV2	ENSG00000160293|ENSG00000182979		1	
VAV3	ENSG00000167325		1	
WDFY3	ENSG00000163625		1	

WDR5	ENSG00000196363		1	
WDR81	ENSG00000167716		1	
WDR82	ENSG00000164091		1	
WEE1	ENSG00000166483		1	
WFDC10B	ENSG00000182931		1	
WNT3A	ENSG00000154342		1	
WNT4	ENSG00000162552		1	
WNT7B	ENSG00000188064		1	1
XKR6	ENSG00000171044		1	
XPO6	ENSG00000169180|ENSG00000182473		1	
YIPF1	ENSG00000058799		1	
YSK4	ENSG00000176601		1	
YTHDF1	ENSG00000149658		1	
YY1	ENSG00000100811		1	
ZBED3	ENSG00000132846		1	
ZBTB26	ENSG00000171448		1	
ZFAND3	ENSG00000156639		1	
ZFPM1	ENSG00000179588		1	
ZHX2	ENSG00000178764		1	
ZNF107	ENSG00000196247		1	
ZNF205	ENSG00000122386		1	
ZNF221	ENSG00000159882|ENSG00000186462		1	
ZNF319	ENSG00000166188		1	
ZNF385	ENSG00000161642		1	
ZNF462	ENSG00000148143		1	
ZNF479	ENSG00000185177		1	
ZNF588	ENSG00000196247		1	
ZNF93	ENSG00000213988		1	
ZSCAN1	ENSG00000152467		1	
RNF222	ENSG00000189051	1		
APC	ENSG00000134982	1	1	1
1HA1 1L3K 1PGZ 1PO6 1U1K	ENSG00000196157	1		

CHD4	ENSG00000111642	1	1
MYO1B	ENSG00000128641	1	
PIK3C2G	ENSG00000139144	1	
PDCD10	ENSG00000114209	1	
BMP5	ENSG00000112175	1	
TAF1L	ENSG00000122728	1	
FCRL3	ENSG00000160856	1	
MAX	ENSG00000125952	1	
1AN2 1HLO 1NKP 1NLW 1R0[illegible]	ENSG00000102207	1	
TSPAN13	ENSG00000106537	1	
KIAA0907	ENSG00000132680	1	
SEMA6D	ENSG00000137872	1	
TLK2	ENSG00000146872	1	
TMEM135	ENSG00000166575	1	
DNAJB4	ENSG00000162616	1	
MIB1	ENSG00000101752	1	
HELZ	ENSG00000198265	1	
SETD5	ENSG00000168137	1	
LOC643637	ENSG00000166840	1	
TWISTNB	ENSG00000105849	1	
SMCHD1	ENSG00000101596	1	
PARP15	ENSG00000173200	1	
MYO10	ENSG00000145555	1	
EBAG9	ENSG00000147654	1	
ACACA	ENSG00000132142	1	
TRAPPC4	ENSG00000196655	1	
2J3T 2JSN 2ZMV	ENSG00000108231	1	
GPX5	ENSG00000079782	1	
SPARCL1	ENSG00000152583	1	
TOP2A	ENSG00000131747	1	
USP20	ENSG00000136878	1	
SFRS16	ENSG00000104859	1	

CCDC123	ENSG00000121289	1	
	ENSG00000184651	1	
PMS2CL	ENSG00000187953	1	
MYC	ENSG00000136997	1	1
HIST1H3F	ENSG00000112727	1	
MOBP	ENSG00000168314	1	
MGEA5	ENSG00000198408	1	
NTAN1	ENSG00000157045	1	
SH3KBP1	ENSG00000147010	1	
UBR5	ENSG00000104517	1	
PLXDC1	ENSG00000161381	1	
CCT7	ENSG00000135624	1	
ADAMTS6	ENSG00000049192	1	
GNS	ENSG00000135677	1	
REST	ENSG00000084093	1	
OR13A1	ENSG00000172678	1	
SART1	ENSG00000175467	1	
CCBL2	ENSG00000137944	1	
TFAM	ENSG00000108064	1	
NPHP3	ENSG00000113971	1	
CLEC4D	ENSG00000166527	1	
MMD	ENSG00000108960	1	
ALS2CR8	ENSG00000138380	1	
NEDD8	ENSG00000129559	1	
1NDD 1R4M 1R4N 1XT9 2BKI	ENSG00000116701	1	
ASF1A	ENSG00000111875	1	
CTDSPL2	ENSG00000137770	1	
CDC2L6	ENSG00000155111	1	
KIAA1239	ENSG00000174145	1	
AC010542.2	ENSG00000187155	1	
STK36	ENSG00000163482	1	
USP14	ENSG00000101557	1	

C6orf114	ENSG00000187461	1
ZNF552	ENSG00000178935	1
DDR2	ENSG00000162733	1
RCL1	ENSG00000120158	1
NDEL1	ENSG00000166579	1
RALGAPA2	ENSG00000188559	1
ARHGDIB	ENSG00000111348	1
TTLL1	ENSG00000100271	1
ZNF680	ENSG00000173041	1
SERPINA6	ENSG00000170099	1
SPRED1	ENSG00000166068	1
LRTM2	ENSG00000166159	1
KCMF1	ENSG00000176407	1
F11	ENSG00000088926	1
URB2	ENSG00000135763	1
FAIM	ENSG00000158234	1
CXorf64	ENSG00000183631	1
ENSG00000152994	ENSG00000152994	1
CCDC65	ENSG00000139537	1
	ENSG00000178225	1
COPG2	ENSG00000158623	1
TSR1	ENSG00000167721	1
	ENSG00000183300	1
GOLIM4	ENSG00000173905	1
HNRPC	ENSG00000092199	1
P2RX7	ENSG00000089041	1
ARHGAP20	ENSG00000137727	1
ABCA5	ENSG00000154265	1
CNTNAP3B	ENSG00000106714	1
C1orf103	ENSG00000121931	1
MARCH1	ENSG00000145416	1
C3orf17	ENSG00000163608	1

ALPK1	ENSG00000073331	1
BIRC8	ENSG00000180152	1
FOLR1	ENSG00000110195	1
SLC47A1	ENSG00000142494	1
STRAP	ENSG00000023734	1
COLEC12	ENSG00000158270	1
ALS2CR4	ENSG00000155755	1
CARM1	ENSG00000142453	1
FMO3	ENSG00000007933	1
C5orf28	ENSG00000151881	1
SHROOM4	ENSG00000158352	1
TFB1M	ENSG00000029639	1
TRIP12	ENSG00000153827	1
ARNTL2	ENSG00000029153	1
	ENSG00000183465	1
MRPS14	ENSG00000120333	1
PARS2	ENSG00000162396	1
ADAMTS7	ENSG00000136378	1
STAT1	ENSG00000115415	1
C8orf76	ENSG00000189376	1
IL20	ENSG00000162891	1
CXorf22	ENSG00000165164	1
AKAP8	ENSG00000105127	1
TNFRSF10B	ENSG00000120889	1
RNF2	ENSG00000121481	1
2H0D 3H8H	ENSG00000168152	1
KIAA1680	ENSG00000184305	1
FGD5	ENSG00000154783	1
C4orf3	ENSG00000164096	1
LOC731797	ENSG00000179066	1
OGN	ENSG00000106809	1
C4orf50	ENSG00000181215	1

	ENSG00000183096	1	
CAP1	ENSG00000131236	1	
PAN3	ENSG00000152520	1	
CCDC45	ENSG00000141325	1	
XRCC4	ENSG00000152422	1	
1FU1 1IK9 3II6	ENSG00000184639	1	
MRPL17	ENSG00000158042	1	
ZMYM3	ENSG00000147130	1	
CNTN1	ENSG00000018236	1	
2EE2	ENSG00000155324	1	
STX7	ENSG00000079950	1	
IQCK	ENSG00000174628	1	
C2orf4P	ENSG00000179408	1	
ENAM	ENSG00000132464	1	
SULT1C2	ENSG00000198203	1	
3BFX	ENSG00000196433	1	
	ENSG00000197398	1	
PTGDR	ENSG00000168229	1	1
SLC4A4	ENSG00000080493	1	
TNKS	ENSG00000173273	1	
SFRS15	ENSG00000156304	1	
U2AF1L4	ENSG00000161265	1	1
SSX8	ENSG00000157965	1	
EDEM2	ENSG00000088298	1	
OR51I1	ENSG00000167359	1	
PHF8	ENSG00000172943	1	
2WWU 3K3N 3K3O 3KV4	ENSG00000159100	1	
PLEKHB1	ENSG00000021300	1	
	ENSG00000116708	1	
GLMN	ENSG00000174842	1	
C12orf64	ENSG00000165899	1	
CWF19L2	ENSG00000152404	1	

SYT1	ENSG00000067715	1
TFPI2	ENSG00000105825	1
ZHX3	ENSG00000174306	1
TMEM69	ENSG00000159596	1
NUDT21	ENSG00000167005	1
LPIN2	ENSG00000101577	1
SLC9A8	ENSG00000197818	1
VAPA	ENSG00000101558	1
AP1AR	ENSG00000138660	1
C3orf55	ENSG00000174899	1
RIF1	ENSG00000080345	1
PRTFDC1	ENSG00000099256	1
2JBH	ENSG00000188436	1
PABPC1	ENSG00000070756	1
BMPER	ENSG00000164619	1
FLJ16165	ENSG00000183760	1
POTEG	ENSG00000187537	1
NOL8	ENSG00000198000	1
BACE2	ENSG00000182240	1
2EWY	ENSG00000143742	1
LYPLAL1	ENSG00000143353	1
OTOG	ENSG00000188162	1
TFB2M	ENSG00000162851	1
EIF1B	ENSG00000114784	1
OPRM1	ENSG00000112038	1
IPP	ENSG00000197429	1
KIAA1841	ENSG00000162929	1
BTG3	ENSG00000154640	1
CUL3	ENSG00000036257	1
TEAD1	ENSG00000187079	1
BIRC2	ENSG00000110330	1
CETN1	ENSG00000177143	1

GKAP1	ENSG00000165113	1
C7orf48	ENSG00000157778	1
SESN1	ENSG00000080546	1
NPHS2	ENSG00000116218	1
LOC729515	ENSG00000122339	1
CRAMP1L	ENSG00000007545	1
	ENSG00000180283	1
ATP1A4	ENSG00000132681	1
ANK2	ENSG00000145362	1
	ENSG00000188974	1
LOC732032	ENSG00000178934	1
1BKZ 2GAL 3GAL 4GAL 5GAL	ENSG00000177932	1
ACOX2	ENSG00000168306	1
	ENSG00000180715	1
HOXC9	ENSG00000180806	1
MARS	ENSG00000166986	1
NCRNA00086	ENSG00000178947	1
TMEM185B	ENSG00000179724	1
PKP2	ENSG00000057294	1
CMTM1	ENSG00000089505	1
PUS7L	ENSG00000129317	1
ENOX2	ENSG00000165675	1
PUF60	ENSG00000179950	1
CCT6B	ENSG00000132141	1
SNX11	ENSG00000002919	1
MESDC2	ENSG00000117899	1
DLX1	ENSG00000144355	1
TMEM207	ENSG00000198398	1
HERPUD2	ENSG00000122557	1
MARCH6	ENSG00000145495	1
G3BP2	ENSG00000138757	1
CCZ1	ENSG00000146574	1

TBXAS1	ENSG00000059377	1
ANKRD16	ENSG00000134461	1
IL17RA	ENSG00000177663	1
ATP12A	ENSG00000075673	1
ZNF221	ENSG00000018607	1
KPNA4	ENSG00000186432	1
WISP2	ENSG00000064205	1
C3orf63	ENSG00000163946	1
SPRED2	ENSG00000198369	1
2JP2	ENSG00000198526	1
FGFR1	ENSG00000077782	1
SPG20	ENSG00000133104	1
ANAPC5	ENSG00000089053	1
MGAM	ENSG00000179087	1
2QLY 2QMJ 3CTT 3L4T 3L4U :	ENSG00000182512	1
TMEM50B	ENSG00000142188	1
BIRC4	ENSG00000101966	1
MKNK2	ENSG00000099875	1
ZNF101	ENSG00000181896	1
SSTR2	ENSG00000180616	1
DYNC1LI2	ENSG00000135720	1
	ENSG00000178446	1
GGNBP2	ENSG00000005955	1
CRBN	ENSG00000113851	1
C2orf84	ENSG00000186453	1
CAPN1	ENSG00000014216	1
STAT3	ENSG00000168610	1
IL2RG	ENSG00000147168	1
TXNDC9	ENSG00000115514	1
IFNE	ENSG00000184995	1
	ENSG00000175447	1
PSMA4	ENSG00000041357	1

ST6GAL1	ENSG00000073849	1
OTUD6B	ENSG00000155100	1
WBP2NL	ENSG00000183066	1
C6orf195	ENSG00000164385	1
CAV3	ENSG00000182533	1
METAP2	ENSG00000111142	1
1B59 1B6A 1BN5 1BOA 1KQ0	ENSG00000170419	1
COX4I1	ENSG00000131143	1
PSMD12	ENSG00000197170	1
IMPG2	ENSG00000081148	1
LECT1	ENSG00000136110	1
ENPP2	ENSG00000136960	1
	ENSG00000145268	1
ERV3	ENSG00000152926	1
GPSM2	ENSG00000121957	1
ST6GAL2	ENSG00000144057	1
SPRY2	ENSG00000136158	1
USP47	ENSG00000170242	1
EFHC1	ENSG00000096093	1
GPR22	ENSG00000172209	1
OR2AJ1	ENSG00000177275	1
C6orf150	ENSG00000164430	1
PFKFB2	ENSG00000123836	1
SPC25	ENSG00000152253	1
2VE7	ENSG00000137259	1
2CV5	ENSG00000137288	1
HIST3H2BB	ENSG00000197435	1
LOC441177	ENSG00000176424	1
PROX1	ENSG00000117707	1
C4orf6	ENSG00000082929	1
HNRNPH3	ENSG00000096746	1
SPOCK1	ENSG00000152377	1

MAD2L1BP	ENSG00000124688	1
ACLY	ENSG00000131473	1
FAHD2B	ENSG00000144199	1
TBL1X	ENSG00000101849	1
CCDC104	ENSG00000163001	1
CHRNA3	ENSG00000080644	1
RIPK3	ENSG00000129465	1
CLRN3	ENSG00000180745	1
MCM8	ENSG00000125885	1
FAM193A	ENSG00000125386	1
TMEM65	ENSG00000164983	1
FBXO4	ENSG00000151876	1
DYNLT1	ENSG00000146425	1
KIF6	ENSG00000164627	1
SSR3	ENSG00000114850	1
PABPC3	ENSG00000151846	1
SUSD1	ENSG00000106868	1
	ENSG00000197737	1
TXNDC16	ENSG00000087301	1
MLL5	ENSG00000005483	1
GPBP1	ENSG00000062194	1
PLEKHA8	ENSG00000106086	1
LOC442582	ENSG00000160828	1
PARP9	ENSG00000138496	1
	ENSG00000198294	1
BXDC2	ENSG00000113460	1
SERPIND1	ENSG00000099937	1
PTX3	ENSG00000163661	1
NDST2	ENSG00000166507	1
ZNF222	ENSG00000159885	1
KIAA0317	ENSG00000119682	1
STK33	ENSG00000130413	1

NUCB2	ENSG00000070081	1		
HPRT1	ENSG00000165704	1		
TSPAN18	ENSG00000157570	1		
DUSP6	ENSG00000139318	1		
BCDIN3	ENSG00000146834	1		
GBA	ENSG00000177628	1		
ZNF292	ENSG00000188994	1		
1X3C	ENSG00000109255	1		
SERBP1	ENSG00000142864	1		
SLC15A1	ENSG00000088386	1		1
FXR1	ENSG00000114416	1		
PKIG	ENSG00000168734	1		
TCEAL1	ENSG00000172465	1		
LDLR	ENSG00000130164	1		
1AJJ 1D2J 1F5Y 1F8Z 1HJ7 1H	ENSG00000163635	1		
PIKFYVE	ENSG00000115020	1		
ITPR1	ENSG00000150995	1		
DNMT1	ENSG00000130816	1	1	
BBS9	ENSG00000122507	1		
SCD5	ENSG00000145284	1		1
TDRD6	ENSG00000180113	1		
HMG20A	ENSG00000140382	1		
TAF5L	ENSG00000135801	1		
SLC17A1	ENSG00000124568	1		
NDUFA6	ENSG00000184983	1		
VTI1A	ENSG00000151532	1		
ZEB2	ENSG00000169554	1		
C7orf41	ENSG00000180354	1		
LAPTM4B	ENSG00000104341	1		
DUS1L	ENSG00000169718	1		
UNC93A	ENSG00000112494	1		
AC009237.8	ENSG00000168992	1		

DNASE1	ENSG00000126594	1
CNBD1	ENSG00000176571	1
GAB1	ENSG00000109458	1
MRGPRX1	ENSG00000170255	1
ACBD4	ENSG00000181513	1
2WH5	ENSG00000052835	1
TMEM87A	ENSG00000103978	1
SPTBN2	ENSG00000173898	1
RBX1	ENSG00000100387	1
ITGA9	ENSG00000144668	1
TMTC3	ENSG00000139324	1
KIAA1276	ENSG00000047662	1
USP13	ENSG00000058056	1
hCG_2023776	ENSG00000197029	1
AC078953.1	ENSG00000183657	1
ACOT13	ENSG00000112304	1
CNOT10	ENSG00000182973	1
ICA1	ENSG00000003147	1
CCNE1	ENSG00000105173	1
ATP2A2	ENSG00000174437	1
BAALC	ENSG00000164929	1
SLC12A1	ENSG00000074803	1
PRSS12	ENSG00000164099	1
NXPH4	ENSG00000182379	1
ZNF25	ENSG00000175395	1
E2F3	ENSG00000112242	1
C10orf81	ENSG00000148735	1
NALCN	ENSG00000102452	1
DNAJC7	ENSG00000168259	1
PIAS1	ENSG00000033800	1
NXF2B	ENSG00000185945	1
1FQV 1FS1 1FS2 1LDK 1P22 2	ENSG00000187999	1

	ENSG00000137104	1		
LSM12	ENSG00000161654	1		
TMEM20	ENSG00000176273	1		
C14orf147	ENSG00000165389	1		
PLEK2	ENSG00000100558	1		
OR52D1	ENSG00000181609	1		
VKORC1	ENSG00000167397	1		
RFX3	ENSG00000080298	1		
PLEKHG4B	ENSG00000153404	1		
CABLES1	ENSG00000134508	1		
NONO	ENSG00000147140	1		
CENPJ	ENSG00000151849	1		
CUZD1	ENSG00000138161	1		
HIST3H3	ENSG00000168148	1	1	
2V1D	ENSG00000091972	1		
DDX60L	ENSG00000181381	1		
TUG1	ENSG00000182457	1		
TMCO3	ENSG00000150403	1		
ADAMTS20	ENSG00000173157	1		
ZNF286A	ENSG00000187607	1		
UNC80	ENSG00000144406	1		1
TANK	ENSG00000136560	1		
EPOR	ENSG00000187266	1		
SCML2	ENSG00000102098	1		
MTMR2	ENSG00000087053	1		
ANKRD2	ENSG00000165887	1		
HRNR	ENSG00000197915	1		
CYC1	ENSG00000179091	1		
CDYL	ENSG00000153046	1		
LYZL4	ENSG00000157093	1		
STOX1	ENSG00000165730	1		
SH2B1	ENSG00000178188	1		

GAS8	ENSG00000141013	1	
	ENSG00000104762	1	
TESK1	ENSG00000107140	1	
GOLGA4	ENSG00000144674	1	
ZYX	ENSG00000159840	1	
	ENSG00000198096	1	
DHCR7	ENSG00000172893	1	
CDHR1	ENSG00000148600	1	
C12orf60	ENSG00000182993	1	
PLCG1	ENSG00000124181	1	
FAM164A	ENSG00000104427	1	
SMARCAL1	ENSG00000138375	1	
RNF5	ENSG00000183574	1	
GPATCH1	ENSG00000076650	1	
SUSD2	ENSG00000099994	1	
LOC730735	ENSG00000185141	1	
THEG	ENSG00000105549	1	1
EMR3	ENSG00000131355	1	
TNFRSF10D	ENSG00000173530	1	
SPHK1	ENSG00000176170	1	
SLC9A3R1	ENSG00000109062	1	
TAS1R1	ENSG00000173662	1	
PDSS2	ENSG00000164494	1	
AC069235.2	ENSG00000189238	1	
GPR177	ENSG00000116729	1	
HAVCR1	ENSG00000113249	1	
C1orf162	ENSG00000143110	1	
PDZK1IP1	ENSG00000162366	1	
STC1	ENSG00000159167	1	
ZKSCAN3	ENSG00000189298	1	
SRC	ENSG00000197122	1	
IL9R	ENSG00000124334	1	

AP1M1	ENSG00000072958	1
PCDH1	ENSG00000156453	1
CHST12	ENSG00000136213	1
2D9Y 2YRY	ENSG00000143940	1
MYL1	ENSG00000168530	1
HSF4	ENSG00000102878	1
FAM178B	ENSG00000168754	1
SLC26A9	ENSG00000174502	1
SLC2A10	ENSG00000197496	1
ERLIN1	ENSG00000107566	1
GPR88	ENSG00000181656	1
AARS2	ENSG00000124608	1
IFI27	ENSG00000165949	1
RPL39L	ENSG00000163923	1
RYK	ENSG00000163785	1
H6PD	ENSG00000049239	1
PCDHB18	ENSG00000146001	1
C13orf34	ENSG00000136122	1
FAP	ENSG00000078098	1
GJB5	ENSG00000189280	1
LASS3	ENSG00000154227	1
RNF216L	ENSG00000196204	1
GRIK1	ENSG00000171189	1
2ZNS 2ZNT 2ZNU 3FUZ 3FV1	ENSG00000095539	1
HS1BP3	ENSG00000118960	1
OR4N2	ENSG00000176294	1
ALDH16A1	ENSG00000161618	1
MDH1	ENSG00000014641	1
ATP13A2	ENSG00000159363	1
NDUFA11	ENSG00000174886	1
TRIM46	ENSG00000163462	1
TESC	ENSG00000088992	1

NOX1	ENSG00000007952	1
HSD17B7	ENSG00000132196	1
MYO18B	ENSG00000133454	1
	ENSG00000182994	1
RTKN	ENSG00000114993	1
TMEM40	ENSG00000088726	1
OR2C1	ENSG00000168158	1
NFKBIL2	ENSG00000160949	1
ITGB4	ENSG00000132470	1
HYI	ENSG00000178922	1
VPS37A	ENSG00000155975	1
KLHDC3	ENSG00000124702	1
PYROXD2	ENSG00000119943	1
CCDC21	ENSG00000130695	1
ProSAPiP1	ENSG00000088899	1
KIAA0892	ENSG00000129933	1
ADM2	ENSG00000128165	1
SLC44A1	ENSG00000070214	1
TMEM56	ENSG00000152078	1
MTHFSD	ENSG00000103248	1
INTS10	ENSG00000104613	1
ANK1	ENSG00000029534	1
1N11 2YQF 2YVI 3F59 3KBT 3	ENSG00000104886	1
C5orf13	ENSG00000134986	1
TFE3	ENSG00000068323	1
APLN	ENSG00000171388	1
TAS2R7	ENSG00000121377	1
KLC4	ENSG00000137171	1
ANKRD23	ENSG00000163126	1
CPSF3L	ENSG00000127054	1
JARID1D	ENSG00000012817	1
2E6R 2YQE	ENSG00000186259	1

MRGPRF	ENSG00000172935	1
	ENSG00000132786	1
	ENSG00000073341	1
LOC440456	ENSG00000159266	1
TMEM102	ENSG00000181284	1
FKSG2	ENSG00000129699	1
MAPBPIP	ENSG00000116586	1
BEST1	ENSG00000167995	1
OR1D2	ENSG00000184166	1
LPA	ENSG00000198670	1
C1RL	ENSG00000139178	1
LOC100132900	ENSG00000197421	1
PFDN2	ENSG00000143256	1
TTC13	ENSG00000143643	1
ZNF655	ENSG00000197343	1
ANKFY1	ENSG00000185722	1
MSLN	ENSG00000102854	1
ZNF347	ENSG00000197937	1
2EMA 2EMP 2EN4 2ENE 2EN	ENSG00000179335	1
NFRKB	ENSG00000170322	1
CYP3A5	ENSG00000106258	1
C14orf135	ENSG00000126773	1
XXBAC-B444P24.1	ENSG00000161132	1
PCBP4	ENSG00000090097	1
AIFM3	ENSG00000183773	1
ASMTL	ENSG00000169093	1
ZCCHC14	ENSG00000140948	1
SLC39A10	ENSG00000196950	1
EIF1AD	ENSG00000175376	1
ABCC9	ENSG00000069431	1
NR4A1	ENSG00000123358	1
2QW4	ENSG00000137341	1

P2RY2	ENSG00000175591		1
YLPM1	ENSG00000119596		1
LOC374973	ENSG00000186118		1
SFRS1	ENSG00000136450		1
GPR107	ENSG00000148358	1	1
1UG1 1UHC	ENSG00000142789		1
LOC100134713	ENSG00000133597		1
NDOR1	ENSG00000188566		1
GRIN3B	ENSG00000116032		1
PRNP	ENSG00000171867		1
GGT5	ENSG00000099998		1
PI4K2A	ENSG00000155252		1
MANBAL	ENSG00000101363		1
WDR59	ENSG00000103091		1
SELENBP1	ENSG00000143416		1
LOC387758	ENSG00000176971		1
NUP107	ENSG00000111581		1
PRDM4	ENSG00000110851		1
THSD1	ENSG00000136114		1
FRMPD3	ENSG00000147234		1
SLAIN1	ENSG00000139737		1
JAK1	ENSG00000162434		1
SAMD12	ENSG00000177570		1
CALN1	ENSG00000183166		1
LRRC70	ENSG00000186105		1
PHTF2	ENSG00000006576		1
CTNS	ENSG00000040531		1
C3orf21	ENSG00000173950		1
PITX3	ENSG00000107859		1
TRIOBP	ENSG00000100106		1
NIN	ENSG00000100503		1
FOXK1	ENSG00000164916		1

CDKN1A	ENSG00000124762	1	
FOXA1	ENSG00000129514	1	
LEPR	ENSG00000116678	1	
DVL2	ENSG00000004975	1	1
2REY 3CBX 3CBY 3CBZ 3CC0	ENSG00000186316	1	
BIRC3	ENSG00000023445	1	1
HCG1782607	ENSG00000196432	1	
MLLT7	ENSG00000184481	1	
1E17 3L2C	ENSG00000162647	1	
RGPD4	ENSG00000196862	1	
NPY2R	ENSG00000185149	1	
IL34	ENSG00000157368	1	
DNMBP	ENSG00000107554	1	
ZNF593	ENSG00000142684	1	
GDPD2	ENSG00000130055	1	
	ENSG00000175340	1	
CASS4	ENSG00000087589	1	
SCGB1A1	ENSG00000149021	1	
NEFL	ENSG00000104725	1	
CER1	ENSG00000147869	1	
2AF0 2V4Z	ENSG00000168806	1	
MED28	ENSG00000118579	1	
LOC732169	ENSG00000105877	1	
ARMCX3	ENSG00000102401	1	
MTMR9	ENSG00000104643	1	
C2CD3	ENSG00000168014	1	
CTNNA1	ENSG00000044115	1	
1H6G	ENSG00000145626	1	
INTS2	ENSG00000108506	1	
SLC25A42	ENSG00000181035	1	
POLR2L	ENSG00000177700	1	
CCDC94	ENSG00000105248	1	

	ENSG00000175142	1
TRIM42	ENSG00000155890	1
RBL1	ENSG00000080839	1
TGM4	ENSG00000163810	1
LOC388965	ENSG00000182814	1
PSME3	ENSG00000131467	1
TCOF1	ENSG00000070814	1
	ENSG00000184249	1
EBNA1BP2	ENSG00000117395	1
LOC100132146	ENSG00000163828	1
KIAA1539	ENSG00000005238	1
GPAT2	ENSG00000186281	1
SOX30	ENSG00000039600	1
PLA2G4B	ENSG00000168970	1
PDCD7	ENSG00000090470	1
EIF3L	ENSG00000100129	1
CRELD1	ENSG00000163703	1
TDRD5	ENSG00000162782	1
COTL1	ENSG00000103187	1
HARS2	ENSG00000112855	1
	ENSG00000184542	1
GTF2A1	ENSG00000165417	1
TNFSF14	ENSG00000125735	1
FAU	ENSG00000149806	1
TAF6	ENSG00000106290	1
DBP	ENSG00000063176	1
B3GALTL	ENSG00000187676	1
HEYL	ENSG00000163909	1
C2orf43	ENSG00000118961	1
PRKAB1	ENSG00000111725	1
KIR2DL1	ENSG00000125498	1
1IM9 1NKR	ENSG00000168442	1

MED8	ENSG00000159479	1	
RPP38	ENSG00000152464	1	
C14orf153	ENSG00000126214	1	
GOT1	ENSG00000120053	1	
HSP90AB1	ENSG00000096384	1	
SCO1	ENSG00000133028	1	
CCL18	ENSG00000006074	1	
LECT2	ENSG00000145826	1	
ZFP36	ENSG00000128016	1	
PFKL	ENSG00000141959	1	
MMP27	ENSG00000137675	1	
ERMP1	ENSG00000099219	1	
THUMPD2	ENSG00000138050	1	
SH3GL1	ENSG00000141985	1	
AGAP1	ENSG00000157985	1	
RPL7	ENSG00000147604	1	
NKD1	ENSG00000140807	1	
ZSCAN23	ENSG00000187987	1	
SASH1	ENSG00000111961	1	
RPL12	ENSG00000197958	1	
MED6	ENSG00000133997	1	
PCID1	ENSG00000149100	1	
CST9L	ENSG00000101435	1	
ACER2	ENSG00000177076	1	
ACTL7B	ENSG00000148156	1	
C9orf98	ENSG00000165695	1	
CMTM3	ENSG00000140931	1	
AQP5	ENSG00000161798	1	1
RHBDD2	ENSG00000005486	1	
RAB1B	ENSG00000174903	1	
GTF2A2	ENSG00000140307	1	
PDZRN4	ENSG00000165966	1	

C12orf51	ENSG00000173064	1	
LOC100129321	ENSG00000157216	1	
FLJ25476	ENSG00000160094	1	
DONSON	ENSG00000159147	1	
STK16	ENSG00000115661	1	
LOC100287530	ENSG00000171611	1	
IZUMO1	ENSG00000182264	1	
CCNL1	ENSG00000163660	1	
FAM22G	ENSG00000188152	1	
CEP78	ENSG00000148019	1	
	ENSG00000174400	1	
VPS26B	ENSG00000151502	1	
FAM47C	ENSG00000198173	1	
RPS17	ENSG00000182774	1	
DOCK6	ENSG00000130158	1	
KIAA1975	ENSG00000151303	1	
NKIRAS1	ENSG00000197885	1	
CKM	ENSG00000104879	1	
1I0E	ENSG00000161677	1	
NFU1	ENSG00000169599	1	
C10orf137	ENSG00000107938	1	
ROBO4	ENSG00000154133	1	
XYLT2	ENSG00000015532	1	
LOC129607	ENSG00000134326	1	
TM4SF5	ENSG00000142484	1	
MKX	ENSG00000150051	1	
ESR1	ENSG00000091831	1	
POLR2K	ENSG00000147669	1	
CUL1	ENSG00000055130	1	
RPS26P11	ENSG00000196933	1	
AXIN1	ENSG00000103126	1	1
NAE1	ENSG00000159593	1	1

ZRANB1	ENSG00000019995	1	1	
TRIM33	ENSG00000197323	1	1	
LOC645381	ENSG00000196781	1		
CLN5	ENSG00000102805	1	1	
NBEAL1	ENSG00000144426	1		
CTPS	ENSG00000171793	1	1	
SLC5A11	ENSG00000158865	1	1	
DNAJC16	ENSG00000116138	1	1	
NLGN1	ENSG00000169760	1	1	
GLE1	ENSG00000119392	1	1	
LEF1	ENSG00000138795	1		
PYGO2	ENSG00000163348	1	1	
FNDC5	ENSG00000160097	1		
FUNDC2	ENSG00000165775	1	1	
COPB2	ENSG00000138032			1
CDC6	ENSG00000171791			1
SURB7	ENSG00000067606|ENSG00000152944		1	1
KPNA3	ENSG00000102753			1
ARCN1	ENSG00000095139|ENSG00000157654|ENSG00000241978			1
COPA	ENSG00000100364|ENSG00000122218			1
COPB1	ENSG00000129083			1
COPG	ENSG00000181789			1
RPH3A	ENSG00000089169			1
MED15	ENSG00000099917			1
TMEM43	ENSG00000170876			1
GNG8	ENSG00000167414			1
ZC3H18	ENSG00000158545			1
BCL9L	ENSG00000186174		1	1
IPMK	ENSG00000151151			1
GART	ENSG00000159131			1
DTX2	ENSG00000091073			1
SLC25A13	ENSG00000004864			1

DTNB	ENSG00000138101	1
LIPE	ENSG00000079435	1
JMJD7	ENSG00000243708	1
GPC4	ENSG00000141030	1
PMM1	ENSG00000100417	1
NDST3	ENSG00000164100	1
MSL3	ENSG00000005302	1
COPZ1	ENSG00000111481	1
TMEM39A	ENSG00000176142	1
FCHSD1	ENSG00000197948	1
OPALIN	ENSG00000197430	1
SYCE1	ENSG00000171772	1
PNPLA1	ENSG00000180316	1
KRTAP13-3	ENSG00000240432	1
SYT10	ENSG00000110975	1
		1
		1
		1
		1
		1
		1
LRRC37A2	ENSG00000238083	1
TAOK2	ENSG00000149930	1
TGFBR1	ENSG00000128268	1
TACR3	ENSG00000169836	1
VN1R5	ENSG00000197617	1
HSPA5		1
NUBP2		1
ZIC1	ENSG00000118513	1
RYR3	ENSG00000198838	1
CEBPD	ENSG00000221869	1
CEACAM4	ENSG00000105352	1

PIN4	ENSG00000102309			1
PLGLB2				1
1AU1				1
HYAL4	ENSG00000106302			1
MARCH2	ENSG00000099785			1
TEX2	ENSG00000136478			1
FAM59A	ENSG00000141441			1
CDT1	ENSG00000167513			1
HSDL2	ENSG00000119471			1
TRIM47	ENSG00000132481			1
				1
DCUN1D3	ENSG00000188215			1
TMED6				1
				1
SSX6				1
				1
SFTA2				1
				1
				1
				1
APPL2	ENSG00000136044			1
ACVRL1	ENSG00000120437|ENSG00000139567			1
CKMT1B	ENSG00000237289			1
CSK	ENSG00000143933|ENSG00000160014|ENSG00000198668		1	1
FER	ENSG00000151422		1	1
2KK6				1
PCK1	ENSG00000197576			1
RPS6KC1	ENSG00000179057			1
STK38L	ENSG00000211455			1
GRK5	ENSG00000198873			1
LPAR6	ENSG00000139679	1		1
SMO	ENSG00000186818			1

PDLIM3	ENSG00000187889		1
			1
TNXB	ENSG00000168477		1
HOXD12	ENSG00000120738		1
CPT1A	ENSG00000110090		1
HMGCS2	ENSG00000134240		1
LYPLA2	ENSG00000011009|ENSG00000116747		1
MTHFR	ENSG00000169618		1
PCCB	ENSG00000114054		1
POLR2D	ENSG00000144231|ENSG00000159110		1
FOLH1B	ENSG00000134612		1
APOBEC2	ENSG00000112337|ENSG00000124701		1
TULP2	ENSG00000104804		1
HTRA1	ENSG00000166033		1
TNS1	ENSG00000079308	1	1
ARFGEF1	ENSG00000066777|ENSG00000116521		1
LMBR1L			1
WISP3	ENSG00000112761		1
CXCR7			1
USPL1			1
OTUB1	ENSG00000167770		1
UCHL3	ENSG00000118939	1	1
MID1	ENSG00000121634		1
			1
CDC45	ENSG00000093009|ENSG00000182070		1
EIF3A	ENSG00000184226		1
LRRC40			1
KCNE1L	ENSG00000176076		1
P2RX5	ENSG00000083454		1
SNRPN			1
HAS1	ENSG00000105509|ENSG00000134755		1
KRT83	ENSG00000170523		1

SMCP			1
PAEP			1
AKAP8L	ENSG00000011243|ENSG00000214941	1	1
HYAL2	ENSG00000113212		1
SUCLG2	ENSG00000145431		1
RHBDL1	ENSG00000103269		1
SLC24A1	ENSG00000074621		1
XAGE2B	ENSG00000155622|ENSG00000185751		1
KIAA0247	ENSG00000100647		1
GLYAT	ENSG00000149124|ENSG00000169855		1
PLAC1	ENSG00000143379		1
POLI	ENSG00000101751		1
1T3N 1ZET 2ALZ 2DPI 2DPJ 2FLL 2FLN 2FLP 2KHU 2KHW 3EPG 3EPI 3G6V			1
WDTC1	ENSG00000142784|ENSG00000168216		1
LPIN1	ENSG00000134324		1
SULF1	ENSG00000137573|ENSG00000184886		1
BAT2L2	ENSG00000117523|ENSG00000165282		1
PSD3	ENSG00000154102|ENSG00000156011		1
CBX7	ENSG00000203837		1
SCRIB	ENSG00000166348|ENSG00000180900		1
GTPBP4			1
SUN2	ENSG00000100242		1
NGEF	ENSG00000122484		1
GGA1	ENSG00000100083|ENSG00000248512		1
CD207	ENSG00000116031		1
ORMDL1	ENSG00000128699		1
TET2	ENSG00000168769		1
C14orf104	ENSG00000165506		1
LGI2	ENSG00000153012		1
PCDHG@			1
ZBTB2	ENSG00000181472		1
OVOL2	ENSG00000125850		1

C21orf63	ENSG00000166979		1
FKBP10	ENSG00000141756		1
TOR1AIP2	ENSG00000169905		1
RSRC2	ENSG00000111011		1
C1orf89	ENSG00000132881		1
RPAP3	ENSG00000005175		1
TXNDC15	ENSG00000113621		1
TRMT2B	ENSG00000188917		1
ULBP1	ENSG00000111981		1
ZBTB45	ENSG00000119574		1
ZNF382	ENSG00000161298		1
			1
			1
ABHD15	ENSG00000168792		1
			1
DSEL	ENSG00000171451		1
			1
TTYH2	ENSG00000141540		1
AGAP2	ENSG00000135439		1
DCD	ENSG00000161634		1
ZNF441	ENSG00000197044		1
HMGB4	ENSG00000176256		1
VSTM2L	ENSG00000132821		1
C20orf141	ENSG00000241690		1
POC5	ENSG00000152359		1
			1
ZNF296	ENSG00000170684		1
FAM151B	ENSG00000152380	1	1
THAP8	ENSG00000161277		1
PRSS55			1
MS4A15			1
			1

		1
ZNF660	ENSG00000144792	1
DDX51	ENSG00000185163	1
KRTAP19-6	ENSG00000186925	1
OTOP3	ENSG00000182938	1
TNFAIP8L3	ENSG00000183578	1
C17orf87	ENSG00000161929	1
CCL4L2		1
NUP62	ENSG00000213024	1
2H4D		1
		1
		1
		1
		1
		1
		1
		1
		1
		1
		1
		1
		1
		1
		1
		1
		1
		1
		1
		1
		1
		1
		1
		1

OR4F15	ENSG00000182854	1
		1
COX4NB	ENSG00000131148	1
TLR1	ENSG00000080561	1
ADRBK1	ENSG00000173020	1
GPR31	ENSG00000058866|ENSG00000120436	1
POU3F3	ENSG00000198914	1
PYCR2	ENSG00000166582	1
SRD5A2	ENSG00000049319	1
UBE2I	ENSG00000116062	1
CPN1	ENSG00000120054	1
GBP1		1
NRAS	ENSG00000213281	1
PHEX	ENSG00000102174	1
PLA2G7	ENSG00000163273	1
RIT2	ENSG00000113520	1
WARS	ENSG00000139505|ENSG00000140105	1
TAPBP	ENSG00000084764	1
1WYO 3CO1		1
TUBG1	ENSG00000131462	1
PRIC285	ENSG00000130589	1
CYTH2	ENSG00000105443	1
EDAR	ENSG00000124067	1
LILRB2	ENSG00000164610	1
MS4A6A	ENSG00000110077	1
UNC5C	ENSG00000204304|ENSG00000206315|ENSG00000224952|ENSG00000225987|	1
CD5	ENSG00000110448	1
USP24		1
		1
STAT5A	ENSG00000178573	1
TRIP4	ENSG00000131238	1
SCARF2	ENSG00000244486	1

ECM1	ENSG00000143369			1
NPHP4	ENSG00000131697	1		1
AQP2	ENSG00000129221|ENSG00000167580			1
AQP3	ENSG00000160224|ENSG00000165272			1
KCNS3	ENSG00000170745			1
DES				1
HMGN1	ENSG00000205581			1
HOXD3	ENSG00000162591			1
GSTM4	ENSG00000183914			1
KCNJ16	ENSG00000131781|ENSG00000153822			1
ABCD4	ENSG00000119688			1
SLC1A6	ENSG00000105143|ENSG00000133424			1
PPT2	ENSG00000168452			1
1YDE				1
JOSD1	ENSG00000100221			1
TSPAN1	ENSG00000117472			1
SEC23A	ENSG00000198626			1
LEPREL2	ENSG00000110811|ENSG00000123453			1
NUP50	ENSG00000093000			1
PLEKHA6	ENSG00000143850	1		1
2D9Y 2YRY				1
CNOT1	ENSG00000169249		1	1
PLCL2	ENSG00000160111			1
KLHL18	ENSG00000114648			1
TTC33	ENSG00000113638			1
TBC1D22A	ENSG00000054611|ENSG00000151576			1
2QFZ				1
CHD5	ENSG00000116254			1
				1
TCTN3	ENSG00000119977			1
TIAM1				1
ATP5S	ENSG00000125375			1

INVS		1
SERP1	ENSG00000171320	1
TMEM97	ENSG00000165312	1
GRHL1		1
CCDC53	ENSG00000120860	1
SUV420H1	ENSG00000110066	1
EGFL7	ENSG00000172889	1
NIP7	ENSG00000132603	1
VCX3A	ENSG00000169059	1
NUP54	ENSG00000138750	1
MPHOSPH8		1
ECHDC2	ENSG00000121310	1
C5orf22	ENSG00000082213	1
TBC1D2	ENSG00000095383	1
TTC27	ENSG00000018699	1
POMGNT1		1
C9orf86		1
RBM22	ENSG00000086589	1
VAC14		1
CACNA2D3	ENSG00000157445	1
EAPP	ENSG00000129518	1
FAM20C		1
PREX1	ENSG00000124126	1
RANBP10	ENSG00000141084	1
		1
CARD9		1
IKZF4	ENSG00000123411	1
VEPH1	ENSG00000197415	1
ELMO3	ENSG00000102890	1
MAP6D1	ENSG00000180834	1
DNAJC22	ENSG00000178401	1
		1

WDR61	ENSG00000140395	1
		1
MAP1LC3A		1
SLC9A7	ENSG00000065923	1
CCDC54	ENSG00000138483	1
FAM83A	ENSG00000147689	1
C21orf70		1
TRIM4	ENSG00000146833	1
GAL3ST3	ENSG00000175229	1
FBXO17	ENSG00000104835	1
GBP4	ENSG00000162654	1
MYOZ3	ENSG00000164591	1
CLNK	ENSG00000109684	1
		1
FAM100A		1
H1FOO	ENSG00000178804	1
SELM		1
MACROD2	ENSG00000172264	1
		1
ZNF280B	ENSG00000198477	1
		1
NIPAL1	ENSG00000163293	1
ZNF595	ENSG00000197701	1
ZNF709	ENSG00000242852	1
XAGE5		1
PSORS1C2	ENSG00000227246|ENSG00000234605	1
NLRP6	ENSG00000174885	1
		1
FOXD4L1	ENSG00000170122	1
		1
ZNF584		1
		1

		1
LPCAT4	ENSG00000176454	1
C19orf75	ENSG00000179213	1
		1
TMSB15B		1
UBE2V1		1
CLEC12B	ENSG00000172322	1
		1
C6orf126	ENSG00000196748	1
2DGR		1
		1
		1
		1
		1
		1
		1
		1
		1
		1
		1
		1
CEACAM22P		1
		1
		1
		1
TARP	ENSG00000211689	1
OR8B2	ENSG00000168234|ENSG00000204293	1
FBXW10	ENSG00000171931	1
CDKL2	ENSG00000241878	1
CEBPE	ENSG00000092067	1
CHRNA4	ENSG00000124508	1
EPC1	ENSG00000120616	1

GIP	ENSG00000113758			1
GPR65	ENSG00000140030			1
RCSD1	ENSG00000198771			1
TGFB3	ENSG00000119699|ENSG00000179087			1
TIAM1	ENSG00000156299			1
RXRB	ENSG00000204231|ENSG00(	1		1
CCDC6	ENSG00000108091			1
CILP				1
CLIC3	ENSG00000160199			1
CSRP2	ENSG00000175183			1
EN2	ENSG00000114859			1
ETF1	ENSG00000120705			1
SSU72	ENSG00000160075			1
C3orf18	ENSG00000088543			1
CSDC2	ENSG00000182107			1
PPAP2C	ENSG00000141934			1
RAD54B	ENSG00000197275			1
SH3BP5	ENSG00000224660			1
SLBP	ENSG00000204301|ENSG00000234876			1
STK33				1
TAS2R14				1
TRPV3				1
SNRNP200	ENSG00000161265		1	1
MECP2	ENSG00000111846			1
ADAM28	ENSG00000042980|ENSG00000157350			1
ADNP	ENSG00000204849			1
BMPR2	ENSG00000165527			1
PRPF6	ENSG00000151718			1
REEP5	ENSG00000144061			1
CD34	ENSG00000174059			1
CDK5R1	ENSG00000157911|ENSG00000176749			1
CDX2	ENSG00000165556			1

VPS13B	ENSG00000132549	1
C1orf61		1
CRYGD	ENSG00000178537	1
CSNK1G1	ENSG00000169118	1
CTDP1	ENSG00000060069	1
FARP2	ENSG00000116473	1
MAGOHB	ENSG00000111196	1
C4orf19	ENSG00000154274	1
EDC3	ENSG00000179151	1
LETM2		1
GNE	ENSG00000159921	1
GSDMD	ENSG00000104518	1
TRIAP1	ENSG00000170855	1
IL24	ENSG00000181856	1
		1
		1
MARK4	ENSG00000007047	1
MC3R	ENSG00000124089	1
ALG13	ENSG00000101901	1
TMEM107	ENSG00000179029	1
CHMP7	ENSG00000147457	1
		1
MVD		1
N4BP2	ENSG00000078177	1
NPY1R	ENSG00000010704	1
SPATA22	ENSG00000141255	1
OSBPL5	ENSG00000021762	1
RNF5		1
SEMA5A	ENSG00000112902	1
		1
SLC16A2	ENSG00000147100	1
SLC4A7	ENSG00000033867	1

SNX1	ENSG00000028528|ENSG00000135363	1
SULT1E1	ENSG00000109193	1
TGIF2LY	ENSG00000176679	1
TNR	ENSG00000116147	1
TRIM52	ENSG00000183718	1
EPDR1	ENSG00000086289	1
UGT8	ENSG00000174607	1
YWHAH	ENSG00000128245	1
2C63 2C74		1
ZNF277	ENSG00000142168	1
ZNF561	ENSG00000171469	1
ABLIM1	ENSG00000099204	1
ADCY1	ENSG00000164742	1
NAAA	ENSG00000138744	1
ATP4A	ENSG00000105675	1
		1
RNF165	ENSG00000141622	1
		1
CCDC3	ENSG00000151468	1
COMMD4	ENSG00000140365	1
HSD17B14	ENSG00000087076	1
CALY		1
FNDC3B	ENSG00000075420	1
TMEM51		1
UFSP2		1
TMEM214	ENSG00000119777	1
SETD6		1
RERGL	ENSG00000111404	1
C22orf15	ENSG00000169314	1
FFAR1	ENSG00000126266	1
HIST1H4C		1
TSC22D2	ENSG00000196428	1

			1
TMEM125	ENSG00000179178		1
FASTKD3	ENSG00000124279		1
MRGPRX3	ENSG00000179826		1
NFAM1	ENSG00000235568		1
NPPB	ENSG00000049860		1
PHC3	ENSG00000173889		1
HDGFL1	ENSG00000249949		1
RAB10	ENSG00000100625		1
RHOG			1
SLC6A16	ENSG00000063127		1
TNS3	ENSG00000136205		1
			1
VAMP5	ENSG00000111786		1
XBP1	ENSG00000100219		1
PELO			1
			1
			1
TRABD			1
			1
			1
			1
HES1		1	
FOXA2	ENSG00000125798	1	
HIST1H2AA	ENSG00000164508	1	
RHOA	ENSG00000198610	1	
SERINC3	ENSG00000132824|ENSG000	1	
CHCHD4	ENSG00000163528	1	
EB9		1	
CASP8		1	
ARL8A	ENSG00000143862	1	
CDC25A	ENSG00000137875	1	

CHMP1		1
CDC42BPB		1
TSPAN3	ENSG00000140391	1
PHB		1
RPN1	ENSG00000198399	1
TMEM113		1
MGA3		1
ELF1	ENSG00000120690	1
TMEM150		1
TMEM165	ENSG00000134851	1
STX5	ENSG00000120289	1
SLC27A6	ENSG00000188739	1
PEX11B		1
SCAP	ENSG00000114650|ENSG000	1
TMEM59	ENSG00000116209	1
SCAP	ENSG00000114650|ENSG000	1
FUBP1	ENSG00000162613	1
HGS	ENSG00000185359	1
FAM57A	ENSG00000167695	1
Brachyury protein		1
THOC4		1
TBPL1	ENSG00000242221	1
SCOTIN		1
CLCN5	ENSG00000171365	1
STCH		1
POFUT1	ENSG00000165511	1
TMEM101	ENSG00000091947	1
UGT3A1	ENSG00000145626	1
SOX7	ENSG00000171056	1
TES-1		1
FUSIP1		1
HIST2H2AB	ENSG00000184270	1

CHSY1	ENSG00000131873	1	
PDNP2		1	
RUNX2	ENSG00000124813	1	
CFHR1	ENSG00000244414	1	
WIPF2	ENSG00000171475	1	
CANT1	ENSG00000171302	1	
SOX17	ENSG00000164736	1	1
EFEMP2	ENSG00000172638	1	
TSKU	ENSG00000163257|ENSG00(	1	
UPK1		1	
ALCAM	ENSG00000069206	1	
PRPF3	ENSG00000117360	1	
RPS3	ENSG00000101384|ENSG00(	1	
C2orf49	ENSG00000135974	1	
ENSG00000112183		1	
BAT3	ENSG00000204463	1	
NOL12	ENSG00000100101	1	
Q59H13		1	
CCDC18	ENSG00000122483	1	
C20orf102		1	
PLOD3		1	
TSC1	ENSG00000165699	1	
IDH2	ENSG00000136628	1	
STX10	ENSG00000104915|ENSG00(	1	
TMEM46		1	
PAX3		1	
SERINC5		1	
SFRS3		1	
CACNA2D2	ENSG00000007402	1	
DLX3	ENSG00000064195	1	
SLC35A3	ENSG00000204033	1	
DERL1	ENSG00000136986	1	

ZNT6		1	
FOXI1	ENSG00000153002|ENSG000	1	
C19orf52	ENSG00000142444	1	
YTHDF2	ENSG00000198492	1	
LACI		1	
SIAT4B		1	
BORG1		1	
RKHD2		1	
OTX1	ENSG00000100292	1	
FBXO16	ENSG00000214050	1	
NOTE: The protein information displayed is the one corresponding to the Gene Name shown.			
MAGOH	ENSG00000162385		1
POU2F1	ENSG00000143190		1
