# Supplementary material for: A functional connectome: regulation of Wnt/TCF-dependent transcription by pairs of pathway activators
Source: Mol Cancer. 2015 Dec 8;14:206. doi: 10.1186/s12943-015-0475-1 (PMC4672529; doi:10.1186/s12943-015-0475-1)
Supplement: Additional file 12: — Supplementary methods. (DOC 213 kb) [file 12943_2015_475_MOESM12_ESM.doc]

Supplementary Methods

Transfection normalisation

Unless otherwise indicated 10 ng/well of CMV-LacZ transfection control plasmid was included in each transfection and luciferase counts were normalised to β-galactosidase activity. If Wnt pathway induction was required, 20 ng of constitutively active LRP6 plasmid was included. If TOPflash/FOPflash reporter activity was to be assayed, 45 ng of each plasmid was added to each well and activity was normalized to TOPflash/vector control activity.

Library Preparation and transfection

The *Xenopus tropicalis* full-length cDNA library used in this study [5] was purchased from MRC Geneservice. Briefly, the library consists of 9,000 individually arrayed cDNA clones and is fully annotated (<http://genomics.nimr.mrc.ac.uk/online/xt-fl-db.html>). The library is constructed in pCS107 vector which permits overexpression from CMV promoter and can also be used to make capped mRNA in vitro. For this study, 9000 plasmid DNA preps, representing the entire library, were pooled 3 to a well for transfections.

As it would have been impractical to measure the concentration of each pool and normalise the concentrations accordingly, a constant volume of pooled DNA was transfected. A 96 well plate of DNA purified using the Wizard SV 96 Plasmid purification kit (Promega) showed that an average of 3 **μ**l contained 70 ng of DNA. ~70ng of pooled DNA was transfected with 20 ng ΔNLRP and 10 ng LacZ expression plasmid. The screen was performed once before selecting wells for deconvolution.

Transfections for the network studies

To allow comparisons to be made between experiments, reporter activity was normalised to the mean activity of 4-6 ΔNLRP positive control wells that were included within each experiment. As a result of these normalisations, ΔNLRP-induced luciferase levels had a defined value of 1.

To identify whether two cDNAs synergised together, 45 ng of each cDNA was co-transfected with 45 ng of every other cDNA identified as a super-activator. As some constitutive activators led to high levels of TCF-dependent transcription, transfected cDNA levels were titrated down to produce the same level of activation of TCF-dependent transcription as was induced by ΔNLRP. 45 ng of each cDNA was co-transfected with a form of β-catenin lacking the N-terminal phosphorylation domain (deltaNbetaCAT, 45 ng/well), the GSK-3 binding domain of Axin (AxinGID, 3 ng/well), a VP16 transactivation domain fused to full length TCF (VP16-TCF, 4 ng/well) and a VP16 transactivation domain fused to the minimal HMG box DNA binding domain of TCF (VP16-minTCF, 4 ng/well). If required, the amount of DNA transfected was made up to 100 ng/well with vector DNA. Where used, small molecule inhibitors were purchased or synthesised in house [6]

Mathematical Analysis of synergy

To identify and investigate synergistic interactions of co-expressed genes, we introduce a metric which quantifies the comparative effect of their interaction on the reporter pathway with that of the constituent genes when expressed individually. We closely follow the methodology of Mani et al. [7] comparing the observed interaction to a null model of no interaction. In [7] a single mutation causes a slower growth rate compared to a wild type - mutations are deleterious. Herein, however, mutations are advantageous by construction in comparison to a control where no cDNA is expressed necessitating a slightly different, albeit analogous, approach.

We compare luminescence values obtained under single and pairwise cDNA transfections comparing them to a background (control) prior to evaluating the metric. The metric itself is then calculated using the means of the observed individual distributions μA and μB and the pairwise μA,B. Those not significantly different from background have their value set to the background value μC. Those with a mean less than the background have their values set to the background value. That is, for some cDNA, labelled A, when expressed in isolation, we obtained a set of reporter activity (luminescence values) XA = {xA,1,...,xA,nA} for nA replicates and similarly XB ={xB,1,...,xB,nB} for some cDNA labelled B with nB replicates. The pairwise activity levels recorded when both cDNAs were co-expressed are thus XA,B ={xA,B,1,...,xA,B,nA,B} for nA,B replicates. Each of these three datasets was compared to a control which is simply a set of m luminescence results, denoted Y = {y1, . . . , ym}, obtained when no cDNA is expressed using a two-tail Kolmogorov Smirnov test at the p < 0.01 significance level. Writing the result of this test as S(Xa,Y) ∈ {0,1} where an outcome of 1 describes the rejection of the null of hypothesis that XA and Y are drawn from the same distribution (i.e. the activity of cDNA labelled A is significantly different from the control), a mean value was assigned to the individual cDNA expression results XA defined as


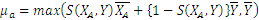
, (1)

where XA and Y are the arithmetic mean of the set of activity values XA and Y. Similar mean values were enumerated for XB, XA,B. Consequently, those cDNA’s whose activity was not significantly different from background (or whose mean activity was less than the mean of the background) were assigned the control mean value, removing low-level anomalies.

The prescribed quantitative measure of interaction, the *synergistic interaction* is [7]


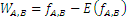
, (2)

where fA,B is the observed fitness of the the co-transfection and E(fA,B) denotes the expected fitness of no interaction. The measure of interaction is the deviation of the observed behaviour compared to a model of anticipated behaviour if there was no interaction between the genes. The co-transfection interaction fitness E(fA,B) is some prescribed function of the individual fitnesses of each individual transfection, termed the Neutrality Function.

As the luminescences can cover a broad range of values (up to 230μC in some cases), it makes sense to use a logarithmic scale and, similar to the relative growth rate used in [7], we prescribe a relative value, (i.e. as an increase compared to background), for the fitness function,


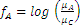
. (3)

For the neutrality function, in this antithetical scenario, it is the Max function which is appropriate,


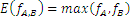
, (4)

in contrast to the more conventional Min function [7]. Substituting the fitness and neutrality into the synergistic interaction equation generates the definition of link weight,


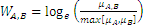
 . (5)

This expression generates a positive link weight for a synergistic interaction in which pairwise co-expressed activity was greater than both individuals (i.e. the added presence of one of the cDNAs enhanced the activity of the other). If only one of the pair activated when individually expressed, any increase above this would constitute a positive interaction. Similarly, if neither cDNA led to an increase in reporter activity above background levels when individually expressed, any reporter activity above background levels upon co-expression would constitute a synergistic interaction.

A negative link weight was prescribed for suppressive interactions in which pairwise activity is less than one of the individuals (i.e. the added presence of one of the cDNAs suppressed the activity of the other). If two cDNAs were co-expressed and either (or both) was capable of inducing reporter activity in- dividually and the pairwise activity was lower than the maximum individual level, a negative link weight was assigned.

Alternative fitness (relative, logarithmic and both) and neutrality (max, min, log, additional, product) functions were considered. Identification of pairwise combinations of regulators using an alternative product rule [8] to identify synergizing pairs (WA,B = μA,B − μAμB ), identified essentially the same set of interactions as identified using equation 2 (454 of the 466 links). This form of analysis was not eventually used to identify the final list of links displayed because the final approach taken produced a non-skewed data distribution centred on zero (an implicit assumption about the data distribution) and used a fitness function that related signal to background (μC). Importantly, the use of the alternative product model would not have altered the gene sets studied in the overlap between functional connectome and the Wnt-protein interaction network (Fig.7).

Derivation of the network visualisation

The network was created using a spring-based method based on the ARF layout [9].

Community Detection on the PIN

To partition the PIN we employed the Louvain method for modularity maximization [10]. This modularity maximisation method was applied to the weighted PIN network (edge weights from the experimental dataset from STRING). The technique of modularity maximisation seeks to identify inherent network structure through determining groups (communities) for which the intra community linking is large in comparison to inter community linking.

Community detection identified 20 different protein complexes from the set of Wnt modulators (Louvain method for modularity maximization [10]; coloured node clusters). It is worth noting that like any other challenging optimization problem it is quite possible to have multiple partitions with very similar energies [1].

**Glossary**

The Wnt ‘Core pathway’: proteins whose function in Wnt signalling have been established in multiple developmental contexts. In this report the following ‘core’ components were studied: LRP6, Dishevelled, Axin, β-catenin and TCF.

ΔNLRP; activated (N-terminal deletion) form of Wnt co-receptor LRP6. Inducers; cDNAs that further increase TCF-dependent transcription induced by ΔNLRP.

Activators; cDNAs that were activate TCF-dependent transcription when expressed alone.

Enhancers; cDNAs that activate TCF-dependent transcription only when co-expressed with other activators or enhancers.

TOPflash; luciferase reporter plasmid with 6X TCF binding sites.

Functional connectome; pattern of synergistic interactions between pathway regulators.

**Supplementary References**

1. Good BH, de Montjoye Y-A, Clauset A: **Performance of modularity maximization in practical contexts.** *Phys Rev E Stat Nonlin Soft Matter Phys* 2010, **81**:046106–046106.

2. Korinek V, Barker N, Morin PJ, van Wichen D, de Weger R, Kinzler KW, Vogelstein B, Clevers H: **Constitutive transcriptional activation by a ß-catenin-Tcf complex in APC-/- colon carcinoma**. *Science* 1997, **275**:1784–1787.

3. Tang W, Dodge M, Gundapaneni D, Michnoff C, Roth M, Lum L: **A genome-wide RNAi screen for Wnt/beta-catenin pathway components identifies unexpected roles for TCF transcription factors in cancer**. *Proc Natl Acad Sci U S A* 2008, **105**:9697–9702.

4. Major MB, Roberts BS, Berndt JD, Marine S, Anastas J, Chung N, Ferrer M, Yi X, Stoick-Cooper CL, Haller von PD, Kategaya L, Chien A, Angers S, MacCoss M, Cleary MA, Arthur WT, Moon RT: **New regulators of Wnt/beta-catenin signaling revealed by integrative molecular screening**. *Sci Signal* 2008, **1**:ra12.

5. Gilchrist MJ, Zorn AM, Voigt J, Smith JC, Papalopulu N, Amaya E: **Defining a large set of full-length clones from a Xenopus tropicalis EST project**. *Developmental Biology* 2004, **271**:498–516.

6. Ewan K, Pajak B, Stubbs M, Todd H, Barbeau O, Quevedo C, Botfield H, Young R, Ruddle R, Samuel L, Battersby A, Raynaud F, Allen N, Wilson S, Latinkic B, Workman P, McDonald E, Blagg J, Aherne W, Dale T: **A useful approach to identify novel small-molecule inhibitors of Wnt-dependent transcription.** *Cancer Res* 2010, **70**:5963–5973.

7. Mani R, St Onge RP, Hartman JL, Giaever G, Roth FP: **Defining genetic interaction.** *Proc Natl Acad Sci U S A* 2008, **105**:3461–3466.

8. Collins SR, Schuldiner M, Krogan NJ, Weissman JS: **A strategy for extracting and analyzing large-scale quantitative epistatic interaction data**. *Genome Biol* 2006, **7**:R63.

9. GEIPEL MM: **SELF-ORGANIZATION APPLIED TO DYNAMIC NETWORK LAYOUT**. *International Journal of Modern Physics C* 2011, **18**:1537–1549.

10. Blondel VD, Guillaume J-L, Lambiotte R, Lefebvre E: **Fast unfolding of communities in large networks**. *J Stat Mech* 2008, **2008**:P10008.
